# RIF1 Is Essential for 53BP1-Dependent Nonhomologous End Joining and Suppression of DNA Double-Strand Break Resection

**DOI:** 10.1016/j.molcel.2013.01.002

**Published:** 2013-03-07

**Authors:** J. Ross Chapman, Patricia Barral, Jean-Baptiste Vannier, Valérie Borel, Martin Steger, Antonia Tomas-Loba, Alessandro A. Sartori, Ian R. Adams, Facundo D. Batista, Simon J. Boulton

**Affiliations:** 1 DNA Damage Response Laboratory, London Research Institute, Cancer Research UK, Clare Hall, South Mimms, London EN6 3LD, UK; 2 Lymphocyte Interaction Group, London Research Institute, Cancer Research UK, 44 Lincoln’s Inn Field, London WC2A 3LY, UK; 3 Institute of Molecular Cancer Research, University of Zurich, Winterthurerstrasse 190, 8057 Zurich, Switzerland; 4 MRC Human Genetics Unit, MRC Institute of Genetics and Molecular Medicine, University of Edinburgh, Western General Hospital, Crewe Road, Edinburgh EH4 2XU, UK

## Abstract

The appropriate execution of DNA double-strand break (DSB) repair is critical for genome stability and tumor avoidance. 53BP1 and BRCA1 directly influence DSB repair pathway choice by regulating 5′ end resection, but how this is achieved remains uncertain. Here we report that *Rif1*^−*/*−^ mice are severely compromised for 53BP1-dependent class switch recombination (CSR) and fusion of dysfunctional telomeres. The inappropriate accumulation of RIF1 at DSBs in S phase is antagonized by BRCA1, and deletion of *Rif1* suppresses toxic nonhomologous end joining (NHEJ) induced by PARP inhibition in *Brca1*-deficient cells. Mechanistically, RIF1 is recruited to DSBs via the N-terminal phospho-SQ/TQ domain of 53BP1, and DSBs generated by ionizing radiation or during CSR are hyperresected in the absence of RIF1. Thus, RIF1 and 53BP1 cooperate to block DSB resection to promote NHEJ in G1, which is antagonized by BRCA1 in S phase to ensure a switch of DSB repair mode to homologous recombination.

## Graphical abstract

**Graphical Abstract:**

**Figure:**
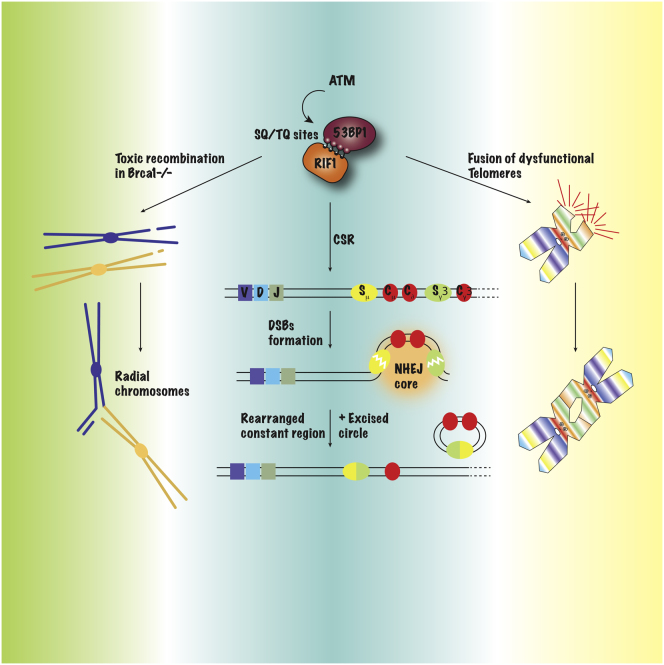

**Highlights:**

► RIF1 is essential for 53BP1-dependent CSR and fusion of dysfunctional telomeres ► BRCA1 antagonizes RIF1 in S phase to prevent error-prone repair by toxic NHEJ ► N-terminal phospho-SQ/TQ domain of 53BP1 interacts with and recruits RIF1 to DSBs ► RIF1 and 53BP1 promote NHEJ in G1 by blocking 5′ end resection of DSBs

## Introduction

DNA double-strand breaks (DSBs) are highly toxic lesions that form when both strands of the DNA duplex are disrupted simultaneously. DSBs arise following exposures to ionizing radiation (IR) and spontaneously as a result of problems encountered during DNA replication that trigger replication fork collapse (Pfeiffer et al., 2000). However, DSBs can also be programmed and are essential during meiosis for promoting exchange between homologous chromosomes to generate genetic diversity and to ensure correct chromosome segregation at meiosis I (Youds and Boulton, 2011). The repair of programmed DSBs is also essential for the production of a full immune repertoire during V(D)J recombination and class switch recombination (CSR) (Stavnezer et al., 2008). DSBs also exist at the end of all linear chromosomes but are normally protected by the telomere and its binding proteins from erroneous repair (de Lange, 2005). Failure to correctly repair DSBs or defects in telomere maintenance have been linked to numerous genetic disorders associated with genome instability, cancer predisposition, accelerated aging, and immune deficiency (Jackson and Bartek, 2009; McKinnon, 2009).

To counter the potential deleterious impact of DSBs, cells have evolved distinct DSB repair pathways, of which nonhomologous end joining (NHEJ) and homologous recombination (HR) are the best understood in eukaryotic cells. NHEJ is a DSB rejoining mechanism that is active throughout the cell cycle but is preferred in G1. NHEJ ensures that broken DSB ends are held in close proximity to permit their direct ligation. As NHEJ operates independently of DNA sequence, it is intrinsically error prone and can drive chromosome translocations by joining DSBs from different parts of the genome (Elliott and Jasin, 2002; Lieber, 2010). In contrast, HR is a largely error-free mechanism of DSB repair that is primarily active in S and G2 cell cycle phases, which requires an intact homologous duplex sequence as a repair template (West, 2003). The initial processing of the DSB ends is a key determinant of DSB repair pathway choice and is tightly regulated during the cell cycle (Symington and Gautier, 2011). In the G1 cell-cycle phase, DSBs are protected to limit DNA end resection, which favors repair by NHEJ. However, upon entry into S phase, DSB end protection is relieved and 5′ end resection is activated to produce DSBs with a 3′ single-stranded overhang, which is the preferred substrate for repair by HR (Symington and Gautier, 2011; West, 2003).

53BP1 is a key DNA repair factor that plays a pivotal role in defining DSB repair pathway choice in G1 and S/G2 cell-cycle phases (Chapman et al., 2012b). Emerging evidence suggests that 53BP1’s association with DSBs in G1 promotes NHEJ by suppressing the inappropriate 5′ resection of DSBs (Bothmer et al., 2010, 2011). As cells enter S phase, the barrier to DSB resection mediated by 53BP1 is alleviated by the action of BRCA1, which drives the removal of 53BP1 from DSBs in S/G2, thus allowing resection and error-free repair by homologous recombination (Bunting et al., 2010; Chapman et al., 2012a). A clear illustration of the antagonistic relationship between 53BP1 and BRCA1 is seen in mouse models, where the embryonic lethality, tumor predisposition, and HR defect of *Brca1*-deficient mice can be rescued by deleting 53BP1 (Bouwman et al., 2010; Bunting et al., 2010; Cao et al., 2009). Based on these observations, it is believed that the defects associated with *Brca1* deficiency reflect an inability to counteract 53BP1-dependent toxic NHEJ in S/G2.

In addition to playing a central role in promoting NHEJ in G1, 53BP1 function is also essential for the establishment of a functional immune system during CSR and is responsible for the erroneous fusion of dysfunctional telomeres (Dimitrova et al., 2008; Manis et al., 2004; Ward et al., 2004). During CSR, activation-induced cytidine deaminase (AID) is essential for the production of DSBs within switch regions located in the immunoglobulin heavy chain (IgH) locus (Neuberger et al., 2003; Pavri and Nussenzweig, 2011). The assembly of antibodies of different class requires that AID-induced DSBs are subject to deletional repair through classical and alternative NHEJ pathways (Stavnezer et al., 2008; Yan et al., 2007). Loss of 53BP1 confers the most severe CSR defect of the DNA repair factors implicated in this process and results in a dramatic decrease in CSR-dependent antibody classes to levels less than 10% of wild-type (WT) (Manis et al., 2004; Ward et al., 2004).

The 53BP1 protein comprises homo-oligomerization and tandem Tudor domains that cooperatively mediate its accumulation at DSB sites via interactions with the H4-K20me2 histone epitope, an extended N terminus containing an abundance of ATM-target SQ/TQ sites, a glycine-arginine rich (GAR) domain permitting PRMT-dependent methylation, and C-terminal BRCT domains that likely mediate phospho-protein interactions (Chapman et al., 2012b). While the GAR and BRCT domains are largely dispensable for 53BP1’s role in DNA repair, the Tudor, oligomerization, and N-terminal SQ/TQ phospho-site domains are essential for 53BP1-dependent inhibition of DSB hyperresection of DSBs induced during CSR and V(D)J recombination (Bothmer et al., 2011; Difilippantonio et al., 2008). Importantly, the same 53BP1 domains have been implicated in driving toxic NHEJ in *Brca1-*deficient cells and for promoting fusion of dysfunctional telomeres (Bothmer et al., 2011; Rai et al., 2010). Of these domains, the function of the N-terminal SQ/TQ phospho-site domain remains the least well understood.

Rif1 was first identified in budding yeast where it negatively regulates telomere length homeostasis via interaction with the C-terminal domain of Rap1 (Hardy et al., 1992; Marcand et al., 1997; Wotton and Shore, 1997). Although there is no clear evidence that the telomere role is conserved in higher eukaryotes, Rif1 has been implicated in a wide variety of other cellular processes, including the intra-S phase checkpoint, timing of replication origin firing, and replication of heterochromatin (Buonomo et al., 2009; Cornacchia et al., 2012; Silverman et al., 2004; Yamazaki et al., 2012). Moreover, *Rif1* gene disruption in mice is lethal, yielding developmental defects in early embryogenesis (Buonomo et al., 2009). Based on analysis of conditional knockout *Rif1*^−*/*−^ cells, it was proposed that the embryonic developmental defects likely reflect important roles during S phase. Indeed, RIF1 is recruited to a subset of stalled replication forks in a manner dependent on both ATR and 53BP1, and in its absence, cells accumulate DNA damage during S phase (Buonomo et al., 2009).

Here, we report that *Rif1*-deficient mice are viable in a CD1 genetic background, yet are immune-compromised due to CSR defects comparable in severity to that observed in 53BP1 null mice. RIF1 is also essential for 53BP1-mediated fusion of dysfunctional telomeres and drives toxic NHEJ in *Brca1*-deficient cells. We further demonstrate that RIF1 functions in the same genetic pathway as 53BP1 and show that the ATM-dependent phosphorylation of 53BP1 promotes 53BP1-RIF1 interactions, recruiting RIF1 to DSB sites. Finally, we find RIF1 suppresses 5′ end resection at DSBs induced by IR and upon CSR. Thus, our findings uncover a key DSB repair role for RIF1 in 53BP1-dependent NHEJ, which has important ramifications for understanding DSB pathway choice and BRCA1’s tumor-suppressive functions.

## Results

### Generation of RIF1-Deficient Mice

RIF1 is the only damage response factor whose recruitment to DSBs strictly depends on 53BP1 (Silverman et al., 2004). However, the function of RIF1 downstream of its recruitment to DSBs by 53BP1 remains unexplored. To investigate the function of RIF1, we generated *Rif1* mutant mice from a Genetrap ESC line (XT278) that harbors an integration event in intron 7 of the *Rif1* gene that removes more than 90% of the RIF1 protein-coding sequence (Figure S1A). Consistent with previous findings (Buonomo et al., 2009), the *Rif1*^XT278^ mutation resulted in embryonic lethality in inbred 129/Ola and outbred MF1 mouse strains (Table S1); E7.5 *Rif1*^XT278/XT278^ homozygous embryos (now referred to as *Rif1*^−*/*−^) exhibited significant developmental retardation (Figure S1B) and produced no live *Rif1*^−*/*−^ offspring (Table S1).

*Rif1*^−*/*−^ E13.5 embryos with developmental features indistinguishable from WT embryos were observed when the *Rif1*^XT278^ allele was crossed into the outbred CD1 strain. Moreover, MEFs derived from *Rif1*^−*/*−^ embryos were viable in culture and expressed no detectable RIF1 protein (Figures S1C and S1D). Strikingly, CD1 *Rif1*^−*/*−^ progeny were also viable, yet only sub-Mendelian numbers of male offspring were observed, indicating that RIF1 deficiency confers female-specific lethality in this background (Table S1). The viability of male *Rif1*^−*/*−^ progeny presented an opportunity to study the impact of RIF1 deficiency in mice.

Surprisingly, viable *Rif1*^−*/*−^ males grow to adulthood, are fertile, and do not exhibit any obvious behavioral or developmental abnormalities. However, the health of some of the *Rif1*^−*/*−^ animals deteriorated rapidly as a result of infections of the commensal bacteria *Staphylococcous xylosus*. Such infections have been reported to affect immune-compromised mice (Gozalo et al., 2010), raising the possibility that RIF1 deficiency might manifest in a suboptimal immune response. The propensity of male *Rif1*^*−/−*^ mice to succumb to infection, coupled to the fact that 53BP1-deficient mice are immune-compromised due to a severe defect in CSR (Manis et al., 2004; Ward et al., 2004), prompted us to examine the immune status of *Rif1*^*−/−*^ mice.

### Normal Lymphocyte Development in *Rif1*^*−/−*^ Mice

To explore the status of the immune system of *Rif1*^*−/−*^ mice, bone marrow B cell populations of *Rif1*^*−/−*^ mice and WT littermates were examined. Notably, no major differences in B cell precursors (pro- and pre-B cells), immature, or mature B cells were evident between WT and *Rif1*^*−/−*^ mice (Figure S2A). Similar analyses of splenocytes revealed no detectable abnormalities in the proportion of B and T cells (Figure S2B), with no significant differences in the percentage of follicular B cells (B220^+^CD21^*−*^CD23^+^) or marginal zone B cells (B220^+^CD21^+^CD23^low^; Figure S2C). Furthermore, no substantial differences were observed in the percentage of immature splenic B cells on the basis of IgM and IgD expression (Figure S2C). Similarly, analyses of T cell populations both in thymus and spleen showed similar proportions of CD4^+^ and CD8^+^ cells in WT and *Rif1*^*−/−*^ mice (Figure S2D). Hence, lymphocyte development appears largely normal in *Rif1*^*−/−*^ mice.

### RIF1 Is Essential for Class Switch Recombination in Mice

**Figure 1:**
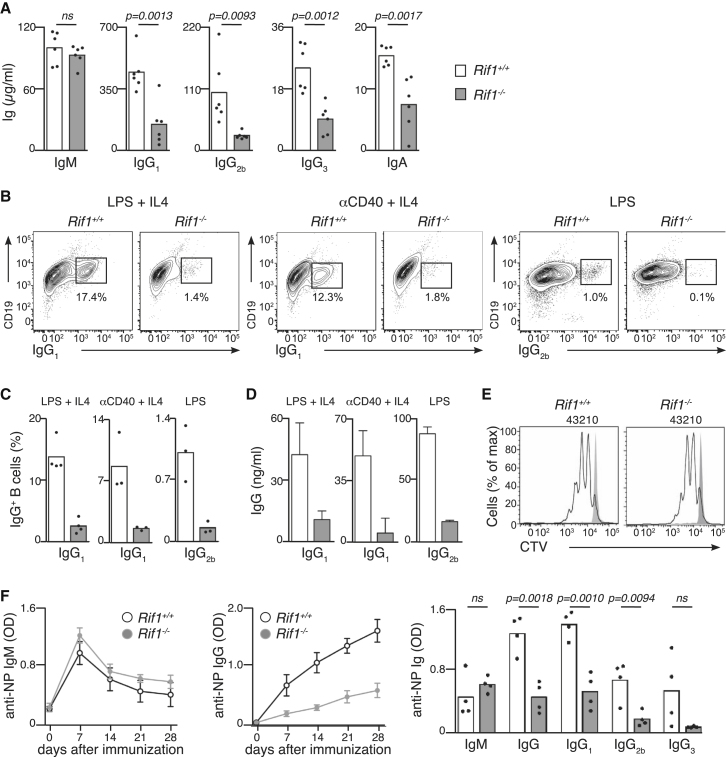
Defective CSR in RIF1*-*Deficient Mice (A) Quantification of immunoglobulins in the serum of *Rif1*^+/+^ and *Rif1*^−/−^ mice. (B–D) Flow cytometry staining (B) and quantifications of surface expression (C) and secreted (D) IgG_1_ and IgG_2b_ in *Rif1*^*+/+*^ and *Rif1*^−/−^ B cells stimulated with LPS or anti-mouse CD40 in the presence or absence of IL-4. (E) B cells were labeled with CTV and cultured for 3 days with anti-mouse CD40 and IL-4 (black line). Grey profile, unstimulated control. Numbers of cell divisions are depicted on the top of the graphs. (F) *Rif1*^+/+^ and *Rif1*^−/−^ mice were immunized with NP-KLH, and NP-specific IgM (left) and IgG (middle) were measured in mice serum at different time points after immunization. Anti-NP-specific antibodies are shown for *Rif1*^+/+^ and *Rif1*^−/−^ mice at 21 days after immunization (right). Error bars are ± SEM. See also Figures S1–S3 and Table S1.

Next we investigated the status of CSR in *Rif1*^*−/−*^ mice by comparing the levels of serum immunoglobulins (Igs) to those of WT animals. As shown in Figure 1A, while no differences were detected in the levels of IgM, the concentrations of IgA and all IgG isotypes were reduced in *Rif1*^*−/−*^ mice, suggesting a possible contribution of RIF1 to CSR. To examine whether such IgG/IgA reductions arise from intrinsic CSR defects, B cells isolated from *Rif1*^*−/−*^ and WT mice were stimulated with lipopolysaccharide (LPS) or anti-mouse CD40 in the presence or absence interleukin 4 (IL-4). CSR proficiency was then assessed by analysis of both cell surface Ig expression and Ig secretion following stimulation (Figures 1B–1D). As indicated, *Rif1*^*−/−*^ cultures exhibited ∼90% reductions in the proportion of IgG positive B cells when compared to WT (Figures 1B and 1C). Similarly, *Rif1*^*−/−*^ B cells were also severely defective in their capacity to secrete IgG, as judged by ELISA (Figure 1D). Importantly, proliferation rates and apoptotic and cell-cycle indices were found to be comparable between WT and *Rif1*^*−/−*^ lymphocytes upon stimulation (Figures 1E, S2E, and S3A). Consistent with a recent report (Cornacchia et al., 2012), replication fork rates and interorigin distances were also comparable between WT and *Rif1*^*−/−*^ cells (Figure S3B).

To further analyze the role of RIF1 in CSR in vivo, *Rif1*^*−/−*^ mice and WT littermates were immunized with the antigen NP-KLH. NP-specific Igs were measured in the serum of these animals over time (Figure 1F). Importantly, while WT and RIF1-deficient mice showed similar levels of NP-specific IgM, *Rif1*^*−/−*^ mice exhibited a severe reduction in the NP-specific IgGs (Figure 1F). To our knowledge the phenotype of *Rif1*^*−/−*^ mice is comparable in severity only to the CSR defect previously reported for 53BP1 deficiency (Manis et al., 2004; Ward et al., 2004). Thus, our data reveal that, like 53BP1, RIF1 is essential for productive CSR in mice.

### Rif1 Promotes NHEJ of Dysfunctional Telomeres

**Figure 2:**
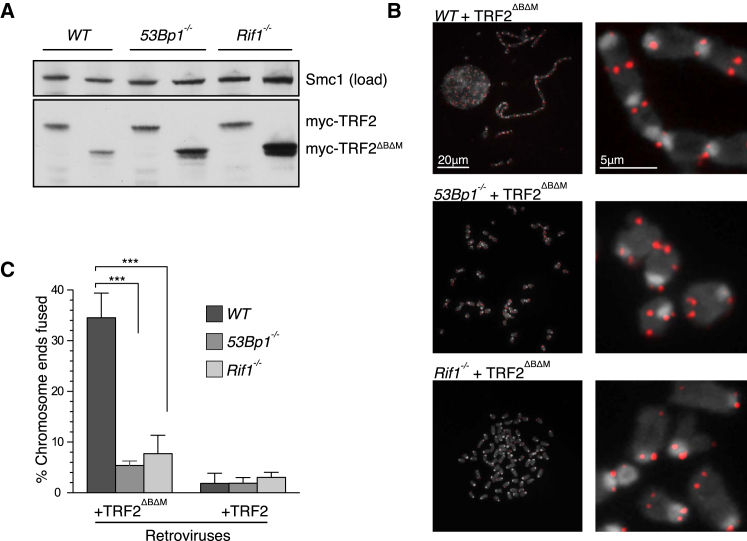
A Requirement for RIF1 in the NHEJ of Dysfunctional Telomeres (A) Western blot showing comparable expression of indicated *Trf2* transgenes between MEF lines of the indicated genotype. (B) Metaphases were analyzed for telomere end-to-end fusions in cells of indicated genotype, 96 hr following retroviral transduction of the indicated TRF2 expression constructs. Right panels show enlargement of corresponding left panel. Telomeric fluorescence in situ hybridization (FISH), red; 4,6-diamidino-2-phenylindole (DAPI), gray. (C) Quantification of chromosome fusions. n = 3,000, 2,000, and 3,000 chromosomes scored per genotype, over two independent experiments. ^∗∗∗^p < 0 .0001 (one-way ANOVA). Error bars are ± SEM.

The similarity of the CSR defect in RIF1- and 53BP1-deficient mice raised the possibility that RIF1 may also function in other 53BP1-dependent processes. In a pathological context, 53BP1 promotes NHEJ between distally positioned telomeres following deprotection by shelterin component disruption (Dimitrova et al., 2008). To determine if RIF1 also facilitates 53BP1-dependent NHEJ events at dysfunctional telomeres, we overexpressed the dominant-negative TRF2^ΔBΔM^ allele of the shelterin component TRF2, which results in telomere deprotection and chromosome end-to-end fusions by NHEJ (Smogorzewska et al., 2002; van Steensel et al., 1998). Metaphases were prepared from WT, *Rif1*^*−/−*^, and *53Bp1*^*−/−*^ MEFs induced to express TRF2^ΔBΔM^ or TRF2 transgenes (Figure 2A) and scored for chromosome end-to-end fusions. In WT cells, overexpression of TRF2^ΔBΔM^, but not TRF2, induced the fusion of 35% of all chromosome ends analyzed (Figures 2B and 2C). Consistent with previous reports, 53BP1 deficiency resulted in a dramatic suppression of end-to-end fusion events at telomeres induced by TRF2^ΔBΔM^ expression (Figures 2B and 2C) (Dimitrova et al., 2008; Rai et al., 2010). Remarkably, RIF1 deficiency also suppressed chromosome joining events, limiting end-to-end fusion to less than 8% of chromosome ends (compared to 35% seen in WT cells and 5% seen in *53Bp1*^*−/−*^ cells; Figures 2B and 2C). These data further suggest that RIF1 functions in 53BP1-dependent NHEJ events.

### BRCA1 Antagonizes RIF1-Dependent NHEJ during S Phase

**Figure 3:**
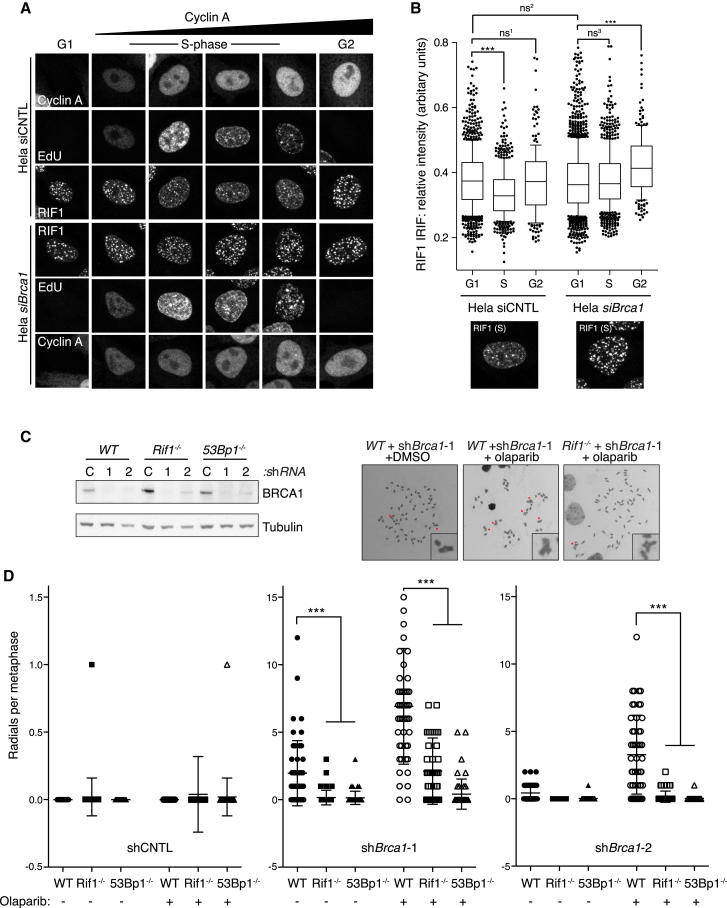
RIF1 Deficiency Suppresses Proliferation and Genome Instability Defects in BRCA1*-*Depleted Cells (A) HeLa cells were treated with the indicated siRNA for 48 hr. Cells were then pulse-labeled with EdU (5 min, 40 μM) immediately before irradiation (5 Gy). One hour following IR, cells were fixed and permeabilized before fluorescent labeling of EdU and counterstaining with indicated antisera. Representative projection images of whole-nuclei 0.5 μm confocal z series are presented. (B) Enlarged S phase images of RIF1 IRIF from (A) and automated quantification of the intensity of RIF1 IRIF in HeLa cells subjected to control or BRCA1-targeting siRNA. n > 140 cells per condition; ^∗∗∗^p < 0.0001, ns^1^ p = 0.514, ns^2^ p = 0.1172, ns^3^ p = 0.1390, Mann-Whitney test. (C) Western blot shows comparable BRCA1-depletion efficiency between MEF lines selected for expression of indicated shRNA constructs. C, control shRNA vector; 1, *Brca1* shRNA vector 1; 2, *Brca1* shRNA vector 2. (D) Radial chromosomes were scored in metaphases prepared from WT, *Rif1*^*−/−*^, and *53Bp1*^*−/−*^ cell lines expressing the indicated shRNA following 16 hr control or olaparib (1 μM) treatments. n = 50 metaphase per condition. Error bars are ± SEM. See also Figure S4.

BRCA1 is believed to antagonize 53BP1-dependent end joining in S phase to promote repair by HR (Bouwman et al., 2010; Bunting et al., 2010, 2012). In agreement with these findings, human 53BP1 and BRCA1 are found to occupy associated yet mutually exclusive chromatin subcompartments at DSB sites, with 53BP1 exclusion from such sites occurring in a BRCA1-dependent manner during S phase (Chapman et al., 2012a). To assess if a similar relationship exists between RIF1 and BRCA1, HeLa cells treated with control and *Brca1* short-interfering RNA (siRNA) were subjected to pulse-labeling with the nucleotide analog EdU immediately before IR, to enable accurate cell-cycle positioning of cells counterstained for RIF1. In addition, Cyclin A counterstaining enabled the discrimination of G1 (EdU-negative) from G2 cell-cycle phases (EdU-negative, Cyclin A-positive). Using this approach we found that in control-depleted cells, RIF1 IR-induced foci (IRIF) are significantly reduced in intensity during S phase when compared to cells in G1 or G2 cell-cycle phases (Figures 3A and 3B). In contrast, the downregulation of RIF1 IRIF in S phase cells was compromised upon BRCA1 depletion, as evident from the intensity of RIF1 IRIF in G1 and S phase cells, which were not significantly different (Figures 3A and 3B). Moreover, the intensity of S and G2 phase RIF1 IRIF were significantly increased in BRCA1-depleted cells when compared with control cells (Figure 3B), and similar results were obtained with a second Brca1 siRNA (Figure S4A). These data indicate RIF1 IRIF are specifically suppressed during S and G2 cell-cycle phases in a BRCA1-dependent manner.

Next, we used lentiviruses to introduce control or two different *Brca1* shRNA expression constructs into MEFs to examine the effect of RIF1 loss on the cellular phenotypes associated with BRCA1 deficiency. Both *Brca1* shRNAs effectively depleted BRCA1 protein in MEFs of all genotypes (Figure 3B). As expected, the expression of either *Brca1* shRNA strongly reduced proliferation rates in WT cells relative to control knockdowns. In contrast, RIF1 or 53BP1 deficiency significantly improved the proliferation of cells expressing each *Brca1* shRNA to similar extents (Figure S4B).

BRCA1*-*deficient cells are hypersensitive to poly(ADP-ribose) polymerase (PARP) inhibitors (PARPi), which trigger the formation of replication-associated DNA damage that requires HR for its resolution (Bryant et al., 2005; Farmer et al., 2005; Helleday, 2011). 53BP1 loss has been shown to suppress both spontaneous and PARPi-induced radial chromosome formation in BRCA1*-*deficient cells (Bunting et al., 2010). To determine if RIF1 also influences the levels of spontaneous and PARPi-induced genomic instability in BRCA1-depleted cells, we analyzed radial chromosomes in metaphase spreads. Radial chromosomes were undetectable in WT untreated control-depleted cells (Figure 3D). In contrast, *Brca1* shRNA induced the formation of spontaneous radial chromosomes in WT cells, which were greatly enhanced by treatment with the PARP inhibitor Olaparib (Figure 3D). Strikingly, RIF1 deficiency exerted a statistically significant suppression of this phenotype, averting the formation of both spontaneous and Olaparib-induced radial chromosomes in *Brca1-*depleted cells to levels similar to that observed in 53BP1-deficent cells (Figure 3D). Thus, like 53BP1, RIF1 contributes to the toxic NHEJ events that drive genome instability in BRCA1-deficient cells.

### Impaired DSB Repair in Rif1-Deficient Cells

**Figure 4:**
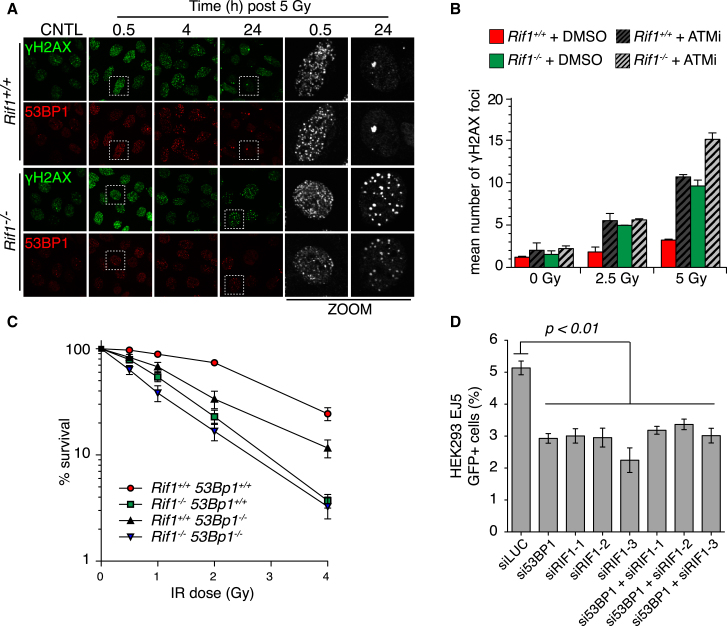
A Role for RIF1 in the Cellular Responses to DSBs (A) WT and *Rif1*^*−/−*^ MEFs were untreated (CNTL) or harvested at indicated times following γ-irradiation before immunostaining with the indicated antisera. Zoom panels correspond to indicated cells in left panels. (B) γH2AX IRIF were scored in cells of indicated genotype 24 hr following mock treatment or IR at the indicated doses. Where indicated, cells were either mock treated (DMSO) or incubated with ATMi (KU55933, 10 μM) from 30 min before irradiation until harvesting. Mean number of γH2AX IRIF per cell is plotted ± SEM. n > 200 cells/condition over two independent experiments. (C) The survival of MEFs of indicated genotype following control or γ-irradiation treatments was assessed by colony survival assay. n = 3 ± SEM. (D) Measurement of NHEJ proficiency in HEK239 EJ5 cells subjected to the indicated siRNAs. n = 3 ± SEM unpaired two-tailed t test. See also Figure S5.

To examine a potential role for RIF1 in IR-induced DSB repair, WT and *Rif1*^*−/−*^ MEFs were harvested at multiple time points following IR treatment and imaged for 53BP1 and the DSB marker γH2AX by indirect immunofluorescence. In both WT and *Rif1*^*−/−*^ cells, γH2AX was apparent as early as 3 min following IR and appeared indistinguishable at earlier time points and up to 8 hr following IR (Figure 4A and data not shown). By 24 hr following IR, the majority of γH2AX foci had been resolved in WT cells; however, multiple γH2AX foci persisted in *Rif1*^*−/−*^ cells at this time point (Figures 4A and 4B). Persistent 53BP1 IRIF were also observed in *Rif1*^*−/−*^ cells, with foci appearing significantly reduced in intensity at all time points when compared to WT cells imaged under identical conditions (Figure 4A). It is notable that the defect in γH2AX resolution in *Rif1*^*−/−*^ cells is similar to that reported in 53BP1-deficient MEFs (Noon et al., 2010; Ward et al., 2006).

To examine the DSB repair defect in more detail, spontaneous and IR-induced γH2AX IRIF were scored in WT and *Rif1*^*−/−*^ cells incubated in the presence and absence of an ATM inhibitor (ATMi) (Figure 4B). Strikingly, WT cells subjected to lower IR doses (2.5 Gy) exhibited γH2AX IRIF levels only marginally higher than control cells, while *Rif1*^*−/−*^ cells showed levels equivalent to WT cells pretreated with ATMi (Figure 4B). Similar correlations in γH2AX IRIF levels between *Rif1*^*−/−*^ and ATMi-treated WT cells were also evident at 5 Gy IR doses (Figure 4B). Moreover, ATMi treatments had minimal impact on γH2AX resolution at lower IR doses in *Rif1*^*−/−*^ cells, suggesting an epistatic relationship between ATM and RIF1 during DSB repair. However, ATMi synergized with *Rif1* deficiency at higher doses, indicating that ATM likely contributes to additional repair functions that are more important in RIF1 deficient cells with higher levels of DNA damage.

We next assessed the relative IR sensitivities of WT, *Rif1*^*−/−*^, *53Bp1*^*−/−*^, and *Rif1*^*−/−*^*53Bp1*^*−/−*^ double-knockout MEFs in colony survival assays. Consistent with siRNA-knockdown experiments in human cells (Silverman et al., 2004), *Rif1*^*−/−*^ MEFs were IR hypersensitive (Figures 4C and S5A), yet this was significantly enhanced relative to that of *53Bp1*^*−/−*^ MEFs (Figure 4C). However, the IR sensitivity *Rif1*^*−/−*^*53Bp1*^*−/−*^ MEFs was not dissimilar from *Rif1*^*−/−*^ cells (Figure 4C), indicating that although 53BP1 and RIF1 likely function in the same pathway during DSB repair, RIF1 may have additional roles in DNA damage responses, consistent with previous observations (Buonomo et al., 2009; Xu et al., 2010).

### Impaired NHEJ in Rif1-Depleted Cells

To directly assess if RIF1 functions to promote 53BP1-dependent DSB repair by NHEJ, we employed an established DSB reporter assay in HEK293 cells that measures NHEJ-mediated repair between two tandem I-SceI sites (Bennardo et al., 2008). As expected, siRNA-mediated 53BP1 depletion resulted in a defect in NHEJ-mediated repair (Figures 4D and S5B). Remarkably, RIF1 downregulation with three different RIF1 siRNAs yielded very similar NHEJ defects to 53BP1-depleted cells (Figure 4D). Moreover, codepletion of 53BP1 and RIF1 did not further enhance NHEJ defects over single knockdowns (Figure 4D), strongly suggesting 53BP1 and RIF1 cooperate during NHEJ.

### RIF1 Recruitment to DSB Sites Requires N-Terminal 53BP1 Phospho-Domain

**Figure 5:**
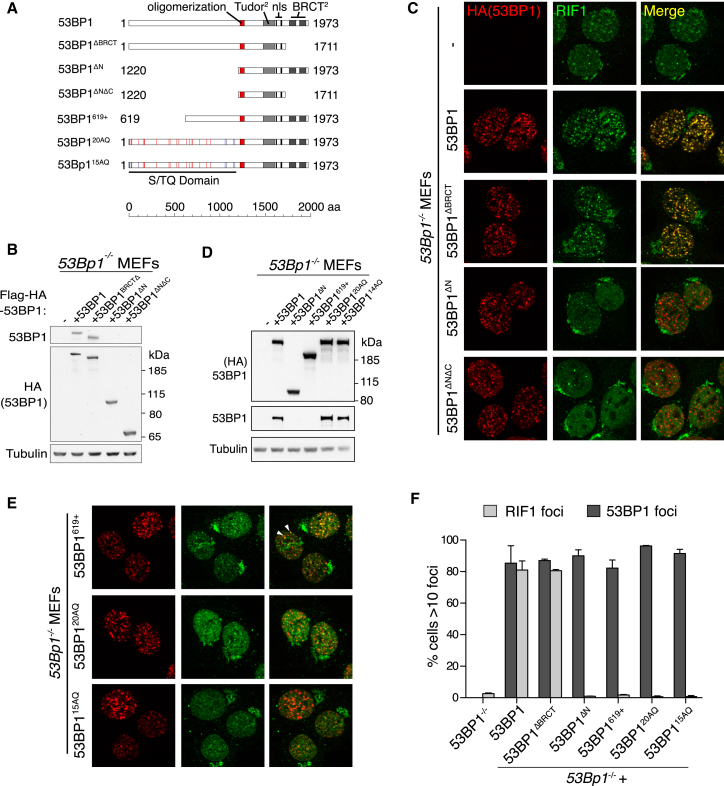
RIF1 Recruitment to DNA Damage Sites Requires Phosphorylation of the of 53BP1 S/TQ Domain (A) Schematic of 53BP1 mutants generated to examine the requirements for RIF1 IRIF. Numbers indicate N- and C-terminal 53BP1 residues for each truncation mutant. Lines indicate relative positions of S/T residues in each S/TQ motif and whether they were either alanine-substituted (red) or left intact (blue) in each respective mutant. (B) Western blot demonstrating comparable expression of hemagglutinin (HA)-tagged 53BP1 mutants analyzed in (C) upon lentivirus-mediated reconstitution in *53Bp1*^*−/−*^ cells. (C) The ability of the indicated 53BP1 mutant to support RIF1 IRIF was examined by indirect immunofluorescence in irradiated cells 1 hr following IR. (D) Western blot of the indicated 53BP1 mutants reconstituted in *53Bp1*^*−/−*^ cells and analyzed in (E) to examine the function of the 53BP1 N terminus and its S/TQ ATM consensus phosphorylation in promoting RIF1 IRIF. (E) Similar to (C) but with the indicated 53BP1 mutants reconstituted in *53Bp1*^*−/−*^ cells. Arrowheads indicate colocalizing foci. (F) Quantification of RIF1 and 53BP1 IRIF with the indicated 53BP1 mutants reconstituted in *53Bp1*^*−/−*^ cells. n = 2, >140 cells scored per condition ± SEM.

53BP1 contains closely opposed homo-oligomerization and tandem Tudor domains that are both required for its interaction with nucleosomes at DSB sites (Botuyan et al., 2006; Huyen et al., 2004; Zgheib et al., 2009). However, the contribution of these domains, its extended N terminus, and C-terminal BRCT motifs in promoting RIF1 recruitment are not known. To investigate the 53BP1 domain requirements for RIF1 IRIF, we created multiple stably transduced *53Bp1*^*−/−*^ MEF lines, each expressing one of a series of 53BP1 mutants (Figures 5A, 5B, and 5D). *53Bp1*^*−/−*^ MEFs reconstituted with full-length or BRCT-deleted 53BP1 protein fully restored RIF1 IRIF, while a 53BP1 mutant protein N-terminally truncated adjacent to its oligomerization motif (53BP1^ΔN^) or a mutant lacking both the N terminus and BRCT domains (53BP1^ΔNΔC^) could not, despite each readily forming 53BP1 IRIF (Figures 5C and 5F).

The 53BP1 N terminus contains 28 S/TQ motifs, which are known to be phosphorylated by the DSB-responsive kinase ATM following IR. To determine if the ATM phospho-sites in the N terminus of 53BP1 are important for RIF1 IRIF, we reconstituted *53Bp1*^*−/−*^ MEFs with a smaller N-terminal truncation mutant (53BP1^619+^), a 53BP1 mutant comprising alanine substitutions of the 20 most N-terminal S/TQ motifs (53BP1^20AQ^); and a similar mutant but with the five most N-terminal S/TQ motifs spanning the PTIP phospho-interaction motif (Ser25) left intact (53BP1^15AQ^; Figures 5A and 5D) (Munoz et al., 2007). Notably, *53Bp1*^*−/−*^ MEFs reconstituted with 53BP1^619+^ recruited residual RIF1 protein into some IRIF, yet these were substantially reduced in number and intensity (Figures 5E and 5F). Strikingly, *53Bp1*^*−/−*^ MEFs reconstituted with either 53BP1^20AQ^ or 53BP1^15AQ^ mutants failed to recruit RIF1 into IRIF (Figures 5E and 5F).

**Figure 6:**
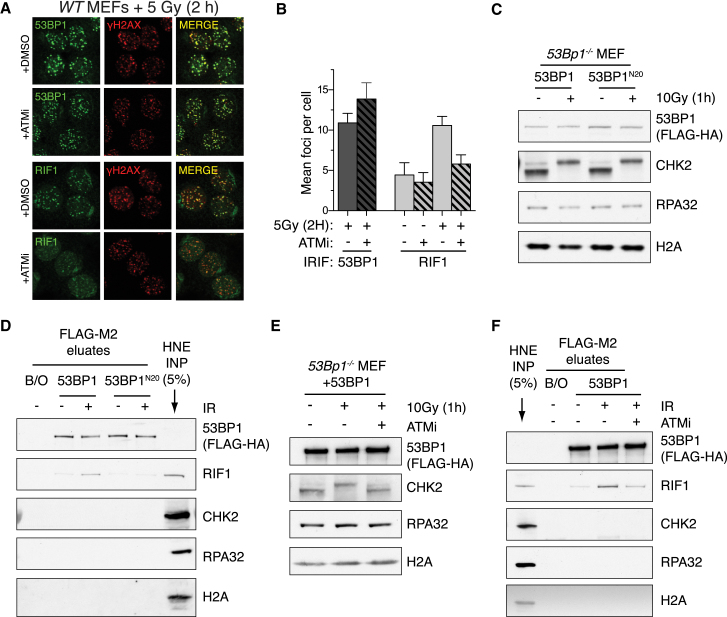
ATM-Dependent Phosphorylation of 53BP1 Promotes RIF1 Interaction (A) Irradiated WT MEFs pretreated with DMSO or ATMi were fixed and immunostained for γH2AX and either RIF1 or 53BP1. (B) Quantification of 53BP1 and RIF1 IRIF in cells prepared as in (A). n > 4 ± SD, >126 cells scored per condition. (C) Immunoblots of whole-cell lysates prepared from *53Bp1*^*−/−*^ MEFs complemented with the indicated constructs as in Figure 5 1 hr following mock or 10 Gy IR. (D) Flag-HA-53BP1 proteins were purified with Flag-M2 beads (Sigma) from lysates in (C) under stringent conditions. Bead complexes were subsequently incubated in nuclear extracts (HNE) before 53BP1-associated proteins were recovered by peptide elution. B/O = beads-only control. (E) Immunoblots of whole-cell lysates of *53Bp1*^*−/−*^ MEFs complemented with WT 53BP1, 1 hr following mock or 15 Gy IR treatments in the presence/absence of ATMi. (F) As in (D), but with FLAG-HA-53BP1 purified from lysates indicated in (E). See also Figure S6.

The requirement for 53BP1 S/TQ phosphorylation in promoting RIF1 recruitment to DSBs prompted us to test the reliance of RIF1 IRIF on ATM kinase activity. Indeed, ATMi treatment severely compromised induction of RIF1 IRIF while inducing a modest increase in 53BP1 IRIF (Figures 6A and 6B). Consistent with previous findings (Buonomo et al., 2009), RIF1 foci apparent in both untreated and ATMi-treated irradiated cells (Figure 6B) were found to be associated with heterochromatin and not DSBs (Figure S6A).

Next, we sought to determine if phosphorylation of 53BP1 by ATM promoted interaction with RIF1. To this end we immunopurified FLAG-HA-53BP1 and its FLAG-HA-53BP1^20AQ^ mutant counterpart from reconstituted *53BP1*^*−/−*^ cells before or after IR treatment (Figures 6C and 6D). 53BP1 proteins were then incubated in untreated nuclear extracts and examined for the ability to bind RIF1. Strikingly, a robust interaction was only observed between RIF1 and 53BP1 purified from IR-treated cells (Figure 6D). In contrast, the 53BP1^20AQ^ mutations compromised RIF1 interaction (Figure 6D) and ATM inhibition also abolished IR-induced interactions between RIF1 and WT 53BP1 (Figures 6E and 6F). Thus, ATM-dependent phosphorylation of 53BP1 is crucial for interaction with and recruitment of RIF1 to DSB sites following DNA damage.

### RIF1 Inhibits DSB Resection at IR-Induced DSBs

**Figure 7:**
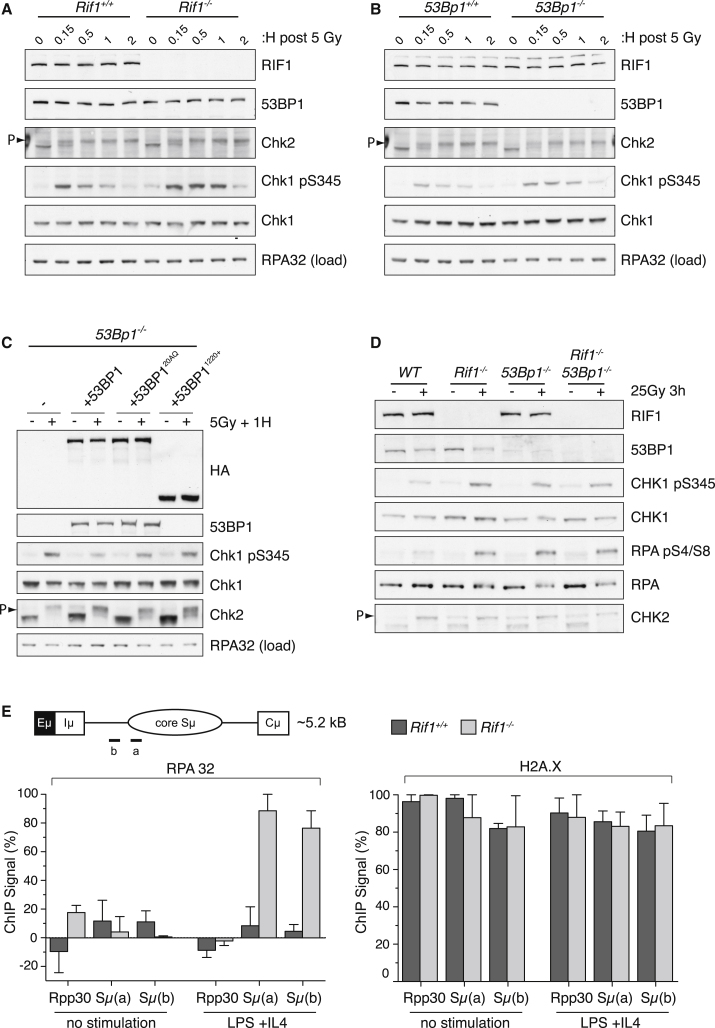
RIF1 Represses DSB Resection Following IR and at the IgH Locus in Stimulated B Cells (A and B) Enhanced ATR signaling in RIF1- and 53BP1*-*deficent cells. Lysates prepared from WT, *Rif1*^*−/−*^, and *53Bp1*^*−/−*^ MEFs, harvested at indicated time points following IR, were immunoblotted with indicated antisera. (C) *53Bp1*^*−/−*^ MEF lines reconstituted with indicated WT and mutant 53BP1 proteins (see also Figures 5A–5D) were examined for IR-induced checkpoint phosphorylation events as in (A) and (B). (D) Lysates prepared from WT, *Rif1*^*−/−*^, *53Bp1*^*−/−*^, and *Rif1*^*−/−*^*53Bp1*^*−/−*^ MEFs harvested 3 hr following IR were immunoblotted with indicated antisera. (E) Aberrant processing of the IgH locus in *Rif1*^*−/−*^ cells. Schematic of IgH Sμ region shows relative positions of qPCR amplicons used in ChIP experiments. A control non-IgH locus (*Rpp30*) was also examined. Resting B cells or those stimulated with LPS and IL-4 (72 hr) were subjected to ChIP experiments with IgG (control), histone H2A.X, and RPA32 monoclonal antisera. Following background subtraction of IgG signals, values were normalized to the DNA input signals, followed by the maximum value in each data set. Mean signals, two independent experiments ± SEM. See also Figure S7.

53BP1 performs a critical role in DSB repair pathway choice by limiting the resection of DSBs induced by IR and during CSR and V(D)J recombination (Bothmer et al., 2010, 2011; Difilippantonio et al., 2008). Given our findings that 53BP1 and RIF1 are both required for NHEJ, we investigated whether RIF1 is also required to prevent 5′-end resection. To this end, we exploited the fact that RPA-ssDNA complexes, resulting from DSB resection, trigger ATR activation (Nam and Cortez, 2011). Furthermore, levels of RPA-ssDNA on 5′-recessed DNA substrates correlate with the degree of Chk1 phosphorylation on Ser345 (pChk1) by ATR (MacDougall et al., 2007). We therefore reasoned that derepression of DSB resection in *Rif1*^*−/−*^ cells might result in enhanced pChk1. Indeed, *Rif1*^*−/−*^ MEFs exhibited significantly elevated levels of pChk1 at all time points examined following IR when compared to WT cells, while cell-cycle profiles and phosphorylation kinetics of the ATM substrate Chk2 appeared comparable in WT and *Rif1*^*−/−*^ MEFs (Figures 7A and S7A). Moreover, IR-induced pChk1 was also enhanced in *53Bp1*^*−/−*^ MEFs (Figure 7B), consistent with its established role in inhibiting resection. Importantly, the enhanced IR-induced pChk1 levels in *53Bp1*^*−/−*^ MEFs was rescued to WT levels by full-length 53BP1, whereas *53Bp1*^*−/−*^ MEFs reconstituted with 53BP1^ΔN^ or 53BP1^20AQ^, which are defective for RIF1 IRIF, failed to suppress enhanced pChk1 levels in *53Bp1*^*−/−*^ cells (Figure 7C). Thus, 53BP1, its N-terminal phospho-SQ/TQ sites, and RIF1 itself all contribute to the suppression of DSB resection.

To further examine a role for RIF1 in blocking DSB resection, we also subjected WT, *Rif1*^*−/−*^, *53Bp1*^*−/−*^, and *Rif1*^*−/−*^*53Bp1*^*−/−*^ MEFs to IR treatment and then analyzed the levels of RPA phosphorylation, an established marker of DSB resection (Sartori et al., 2007). Strikingly, RIF1- and 53BP1-deficient cells exhibited markedly enhanced RPA phosphorylation after IR treatment when compared to WT cells. Furthermore, RPA phosphorylation was not further enhanced in *Rif1*^*−/−*^*53Bp1*^*−/−*^ cells when compared to *Rif1*^*−/−*^ and *53Bp1*^*−/−*^ knockout cells (Figure 7D).

### RIF1 Inhibits DSB Resection at DSBs Induced during CSR

We reasoned that the observed defects in CSR detected in *Rif1*^*−/−*^ lymphocytes might result from a failure to efficiently repress the inappropriate nucleolytic processing of DSBs induced during CSR. Indeed, the failure to switch isotype during CSR in *53Bp1*^*−/−*^ lymphocytes is associated with increased nonproductive short-range recombination events within IgH locus switch (S) regions mediated by repair of aberrantly processed DSBs (Bothmer et al., 2010; Reina-San-Martin et al., 2007). To examine if inappropriate processing of S-regions might also occur in the absence of RIF1, WT and *Rif1*^*−/−*^ lymphocytes were either untreated or stimulated to class switch in vitro and then subjected to chromatin immunoprecipitation (ChIP) for total histone and RPA32. Similar histone ChIP signals were observed between WT and *Rif1*^*−/−*^ lymphocytes at a control locus (Rpp30) and within (Sμ(a)) and proximal (Sμ(b)) to the core Sμ-region (Figure 7E). In contrast, analysis of RPA32 ChIPs from WT and *Rif1*^*−/−*^ lymphocytes induced to undergo CSR revealed striking differences in ChIP signals: *Rif1*^*−/−*^ cells exhibited ∼10-fold increases in RPA32 residency at both Sμ loci when compared to WT, while comparable near background RPA32 signals were evident at the Rpp30 control locus in both cell types (Figures 7E and S7B). Importantly, the induction of RPA32 ChIP signal at IgH loci in RIF1-deficient lymphocytes was only evident upon B cell stimulation; unstimulated cells exhibited near background signals. These data reveal that RPA32 accumulates within the IgH switch region in switching RIF1-deficient cells, indicative of the aberrant resection of CSR-induced DSBs.

## Discussion

RIF1 is one of a few proteins identified to date that requires 53BP1 for its recruitment to DSBs (Silverman et al., 2004). Nevertheless, a role for RIF1 as a potential 53BP1 cofactor has been largely overlooked, in part due to the reported phenotypic differences of murine models of RIF1 and 53BP1 deficiency: RIF1 was previously reported as essential for embryogenesis (Buonomo et al., 2009), whereas *53Bp1*^*−/−*^ animals are viable but radiosensitive and immune-deficient (Manis et al., 2004; Ward et al., 2003, 2004). Moreover, vertebrate RIF1 performs regulatory roles during DNA replication that are not obviously shared with 53BP1 (Buonomo et al., 2009; Cornacchia et al., 2012; Xu et al., 2010; Yamazaki et al., 2012), and RIF1 depletion/disruption was reported to affect gene conversion frequencies to different extents in different cell types (Buonomo et al., 2009; Wang et al., 2009), which was also at odds with a role for RIF1 in promoting 53BP1-dependent NHEJ.

Here we report that *Rif1-*deficient male mice are viable in a specific genetic background. Although *Rif1*^*−/−*^ male mice are superficially normal, they succumb to bacterial infections owing to CSR defects comparable in severity to that previously reported for 53BP1-deficient mice. Indeed, analysis of RIF1*-*deficient MEFs revealed that RIF1 is essential for 53BP1-dependent NHEJ. This assertion is based on our observation that, analogous to 53BP1, unconstrained RIF1 activity both drives toxic repair events in *Brca1-*deficient cells and mediates the NHEJ of dysfunctional telomeres. RIF1*-*deficient cells also exhibit hallmarks of defective DSB repair including IR sensitivity and delayed DSB resolution. Moreover, the fact that *53Bp1*^*−/−*^*Rif1*^*−/−*^ double-knockout MEFs were found to be no more sensitive to IR treatment than the *Rif1*^*−/−*^ single-knockout MEFs suggests that RIF1 and 53BP1 function in the same genetic pathway. The epistatic nature of RIF1, 53BP1, and double siRNA treatments on NHEJ frequency in human cells reinforces this notion. In this light, it is notable that a role for RIF1 during NHEJ was suggested by previous work in the avian DT40 cells. In this setting, RIF1 deficiency was accompanied by 2- to 4-fold increases in gene-targeting frequencies (Xu et al., 2010), a characteristic often used as a measure of HR proficiency in DT40, and whose elevation is characteristic of NHEJ mutants including 53BP1-knockout lines (Nakamura et al., 2006; Yamazoe et al., 2004).

Our analysis also establishes a mechanistic basis for RIF1-53BP1 cooperation. Specifically, we find that the phosphorylation of 53BP1 by ATM stimulates interaction with RIF1, facilitating RIF1 targeting to DSBs. Thus, our findings provide molecular insight to explain the importance of 53BP1 phosphorylation, whose critical role in blocking DSB-resection and promoting NHEJ during CSR and between uncapped telomeres was previously unclear (Bothmer et al., 2011; Rai et al., 2010). Unfortunately, RIF1’s domain architecture offers few clues into how ATM-dependent phosphorylation of 53BP1 actually facilitates RIF1 interactions via phospho- or charge-specific interactions. Nevertheless, as ATM signaling also promotes DSB resection upon 53BP1 disruption (Bothmer et al., 2010; Bunting et al., 2010), its direct stimulation of RIF1 recruitment to DSBs might represent a molecular switch to suppress such activities when 53BP1 is present at DSB sites.

Recent evidence suggests that 53BP1’s ability to establish an effective barrier against DSB resection in G1 is intrinsic to its NHEJ function (Bothmer et al., 2010, 2011; Bunting et al., 2012; Difilippantonio et al., 2008). We find RIF1 is central to this process, its deficiency enhancing ATR checkpoint signaling in response to IR treatments, a feature suggestive of DSB hyperresection and mirrored by *53Bp1*^*−/−*^, *Rif1*^*−/−*^ *53Bp1*^*−/−*^, and complemented cell lines defective in RIF1 recruitment. Finally, RIF1’s role in blocking aberrant DSB processing is substantiated by our findings that abnormally high levels of the ssDNA-binding RPA32 protein accumulate at IgH S-regions in *Rif1*^*−/−*^ B cells upon stimulation. Significantly, similar IgH-specific differences in RPA32 signal were recently reported between WT and *53Bp1*^*−/−*^ B cells in ChIP-sequencing experiments (Bunting et al., 2012; Hakim et al., 2012). Thus our data establish that cooperative inhibition of DSB resection by 53BP1 and RIF1 is crucial for NHEJ.

The function of 53BP1 in blocking DSB resection requires its ability to oligomerize and bind the abundant histone H4 K20me2 epitope (Bothmer et al., 2011). Intriguingly, we found that 53BP1 IRIF are diminished in intensity in *Rif1*^*−/−*^ cells (Figure 4A). Perhaps cooperative interactions between 53BP1 and RIF1 act to stabilize a DSB-proximal chromatin state refractory to access by nucleases and other DNA modifying enzymes, thereby blocking DSB resection over large distances spanning DSB ends. While many genetic observations in yeast attribute resection inhibition largely to the dsDNA end-binding Ku-heterodimer (Ku) (reviewed in Symington and Gautier, 2011), recent evidence in mice indicates that 53BP1 asserts much more of a repressive effect on this activity than Ku (Bunting et al., 2012). Indeed, the fact that 53BP1 binds chromatin (and not DNA ends) suggests that resection may actually be influenced at a distance from DNA ends. Thus, establishment of a 53BP1-RIF1-mediated chromatin barrier in the vicinity of DSBs may ensure DSB end integrity is preserved, favoring NHEJ, and preventing deleterious repair events. In addition to posing a barrier to DSB resection, 53BP1 has also been attributed an ability to increase the subnuclear mobility of dysfunction telomeres, an activity proposed to increase the contact frequency of distally position DSB ends and thus their ligation (Dimitrova et al., 2008). Considering our observed role for Rif1 in promoting telomere end-fusions, it will be intriguing to see if RIF1 also contributes to this process.

Genetic observations in mice (Bothmer et al., 2011; Bunting et al., 2010) led to the proposal that 53BP1 binding to chromatid breaks in BRCA1-deficient cells interferes with HR by blocking resection at the break site, which is required for HR-dependent repair. To counteract an inhibitory activity toward DSB resection in S phase, it was suggested that BRCA1 may act to displace NHEJ factors from replication-associated DSBs, but evidence to support this model was lacking (Bunting et al., 2010). We recently showed that while 53BP1 forms foci at DSBs in G1 and S cell-cycle phases, it is excluded from the core of foci in an S phase and BRCA1-dependent manner (Chapman et al., 2012a). We proposed that the timely removal of 53BP1 from the core of the focus in S phase relieves the barrier to DSB resection, allowing HR dependent repair to proceed. Intriguingly, we now show that BRCA1 is also responsible for the downregulation of RIF1 IRIF in both S and G2 cell-cycle phases, suggesting that while this likely contributes to a switch from NHEJ to HR repair modes during S phase, BRCA1 may also prevent excessive RIF1/53BP1-depedent NHEJ in G2 in a similar manner. Certainly, an improved understanding of how 53BP1 and RIF1 cooperate within their DSB-associated chromatin environment to inhibit resection may be key to elucidating BRCA1’s molecular role in counteracting such activities and defining the mechanisms of DSB repair pathway choice. We anticipate future studies investigating RIF1’s regulatory roles in other nuclear contexts may provide vital clues to understand how it facilitates productive recombination events in lymphocytes and toxic repair events that drive tumorigenesis.

## Experimental Procedures

### Lymphocyte Methods and In Vitro CSR

B cells were purified by negative selection from single-cell suspensions from spleen using magnetic separation B cell isolation kit (Miltenyi Biotec) according to manufacturer’s instructions. Purified cells (purity > 95%) were cultured for 3–6 days at 10^6^ cells/ml in RPMI supplemented with 10% FCS and LPS (10 μg/ml, Sigma), IL-4 (10 ng/ml, R&D), and/or anti-mouse CD40 antibody (5 μg/ml; FGK45, Enzo Life Sciences). For proliferation analyses B cells were labeled with 5 μM Cell Trace Violet (Molecular Probes) in PBS for 15 min at 37°C before culture. For details of antibodies, ELISA, and flow cytometric methods, see Supplemental Information.

### Cell Culture, Transfection, and shRNA

E13.5 MEFs prepared and SV40 LargeT-immortalized by standard procedures were used in all experiments unless otherwise indicated. HeLa and HEK293 cells were transfected with vector or siRNA using XtremeGene-HP (Roche) and siRNA-MAX (Invitrogen) reagents, respectively, according to manufacturers’ instructions. NHEJ reporter assays in HEK293-EJ5 cells were performed as previously described (Bennardo et al., 2008), with minor modifications (see Supplemental Information). *Brca1-*targeting shRNA oligos 1 (target seq: CCAAGAAGAGGATAGTATAAT) and 2 (target seq: GTGCTTCCACACCCTACTTAC) were cloned into pLKO.1-Puro plasmid (Addgene plasmid 10878), and these or a control (empty vector) were used to generate lentivirus to deliver shRNA. Viral supernatants were prepared as described (http://www.addgene.org/plko), and frozen virus was used to transduce MEFs in two sequential infections 12 hr apart, before selection in 2–3 μg/ml puromycin. Shelterin disruption was achieved by retroviral transduction of pLPC-Myc-TRF2 and pLPC-Myc-TRF2^ΔBΔM^ constructs as previously described (van Steensel et al., 1998).

### Immunoprecipitation and Protein Interaction

Flag-HA-53BP1 protein was immunopurified on Flag-M2 agarose (Sigma), from whole-cell lysates prepared as previously described (Chapman and Jackson, 2008): washed in lysis buffer × 2, in TSE-150 (see ChIP in Supplemental Information) × 1, and in TSE-500 × 1 before equilibration in Dignam’s buffer D (20 mM HEPES [pH 7.4], 20% glycerol, 0.1 M KCl, 1 mM EDTA, 0.1 mM EGTA, 1 mM DTT, protease inhibitors). Beads were then incubated in HeLa nuclear extracts (CilBiotech.be) for >1 hr before multiple washes in Buffer D. Immunocomplexes were eluted with triple-Flag peptide. For detailed ChIP methods, see Supplemental Information.

## Supplemental Information

Document S1. Figures S1–S7, Tables S1 and S2, and Supplemental Experimental Procedures
