# Supplementary material for: RIF1 Is Essential for 53BP1-Dependent Nonhomologous End Joining and Suppression of DNA Double-Strand Break Resection
Source: Mol Cell. 2013 Mar 7;49(5):858–71. doi: 10.1016/j.molcel.2013.01.002 (PMC3594748; doi:10.1016/j.molcel.2013.01.002)
Supplement: Document S1. Figures S1–S7, Tables S1 and S2, and Supplemental Experimental Procedures [file mmc1.pdf]

Molecular Cell, Volume *49*

Supplemental Information

RIF1 Is Essential for 53BP1-Dependent Nonhomologous End Joining and Suppression of DNA Double-Strand Break Resection

J. Ross Chapman, Patricia Barral, Jean-Baptiste Vannier, Valérie Borel, Martin Steger, Antonia Tomas-Loba, Alessandro A. Sartori, Ian R. Adams, Facundo D. Batista, and Simon J. Boulton

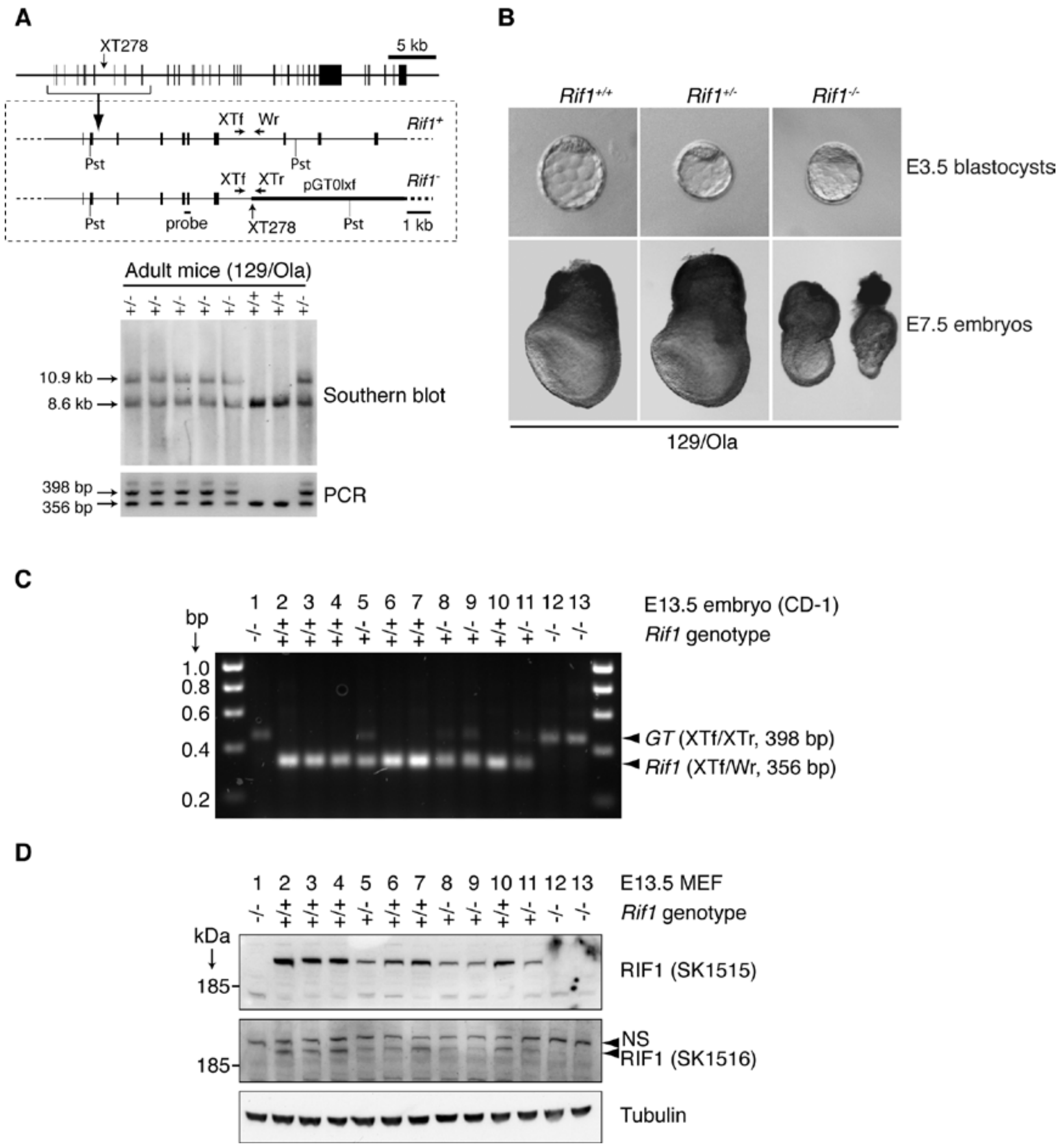

Figure S1 (related to Figure 1). Generation of *Rif1* mutant mice and MEF lines
(**A**) Schematic diagram of the wild-type (Rif1^{+}) and XT278 genetrap (Rif1^{-}) alleles (upper panels). The XT278 genetrap integration site in intron 7 is depicted by an arrow and genetrap vector sequences indicated by the bold line. The PstI restriction sites and the position of the probe used for Southern blotting are shown, as are the XTf, XTr and Wr primer binding sites used for genotyping by PCR. Correct genetrap integration and genotyping strategies were validated in mice born from a Rif1$^{+/-}$ intercross. WT Rif1^{+} and genetrap Rif1^{-} alleles give rise to 8.6 kb and 10.9 kb PstI fragments, respectively (lower panel). (**B**) The Rif1XT278 genetrap allele was crossed into the inbred 129/Ola mouse strain. E7.5 embryos exhibiting embryonic retardation confirmed that Rif1 is essential for embryonic development. (**C**) PCR to genotype mice born from a Rif1$^{+/-}$ intercross generating bands of 398 and 356 bp products for the Rif1^{-} and Rif1^{+} alleles, respectively. (**D**) Embryos from Rif1$^{+/-}$ intercrosses (outbred CD-1 background) were harvested to generate primary embryonic fibroblast (MEF) cultures. Western blotting with two different RIF1 antibodies (SK1515, SK1516) confirmed the loss of detectable RIF1 protein.

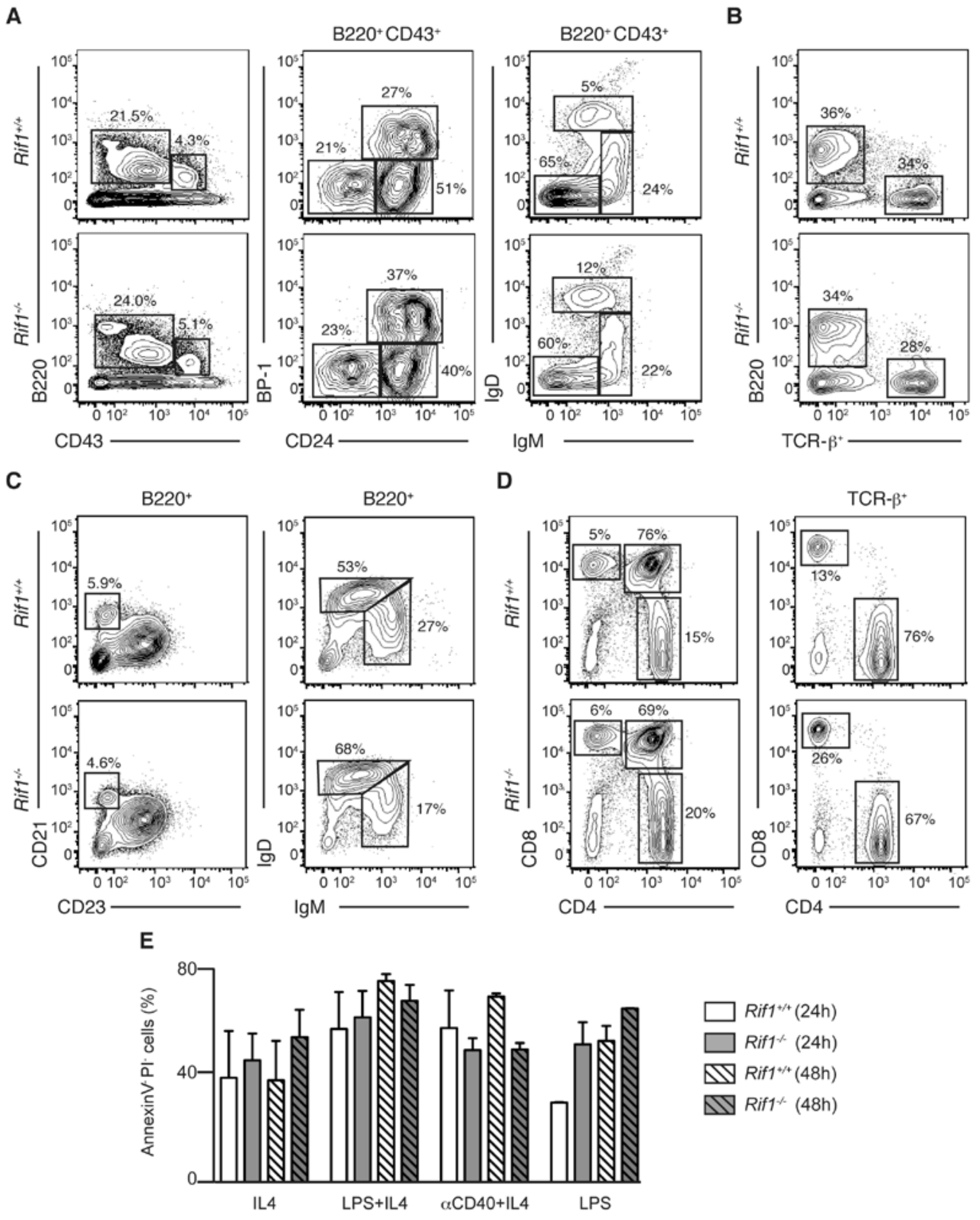

Figure S2 (related to Figure 1). Normal lymphocyte development in Rif1-/- mice Frequency of lymphocyte populations in Rif1+/+ and Rif1-/- mice in bone marrow (A), spleen (B, C and D, right) and thymus (D, left). (**A**) B cell percentages are shown for the different populations in the bone marrow (A-F) as described by Hardy et al (J exp med, 1991) and gated as follows: A, B220+CD43+BP-1-CD24-; B, B220+CD43+BP-1-CD24+; C, B220+CD43+BP-1+CD24+; D, B220+CD43-IgM-IgD-; E, B220+CD43-IgM-IgD+; and F, B220+CD43-IgM+IgD+. (**B**) Percentages of B cells (B220+TCR-b-) and T cells (B220-TCR-b+) in the spleen. (**C**) Subpopulations of B220+ splenocytes have been gated as: marginal zone, (left; B220+ CD23lowCD21high), mature (right; B220+IgDhighIgMlow) and immature (right; B220+ IgDlowIgMhigh) B cells. (**D**) T cell populations were identified in thymus (left) and spleen (right) based on CD4 and CD8 expression. (**E**) Quantification of percentage of Annexin V^- PI^- cells in cultures from RIF-$1^{+/+}$ and RIF-$1^{-/-}$ B cells stimulated during 24 or 48 h with IL4, LPS and IL4, anti-mouse CD40 and IL-4 or LPS. Error bars are ±SEM.

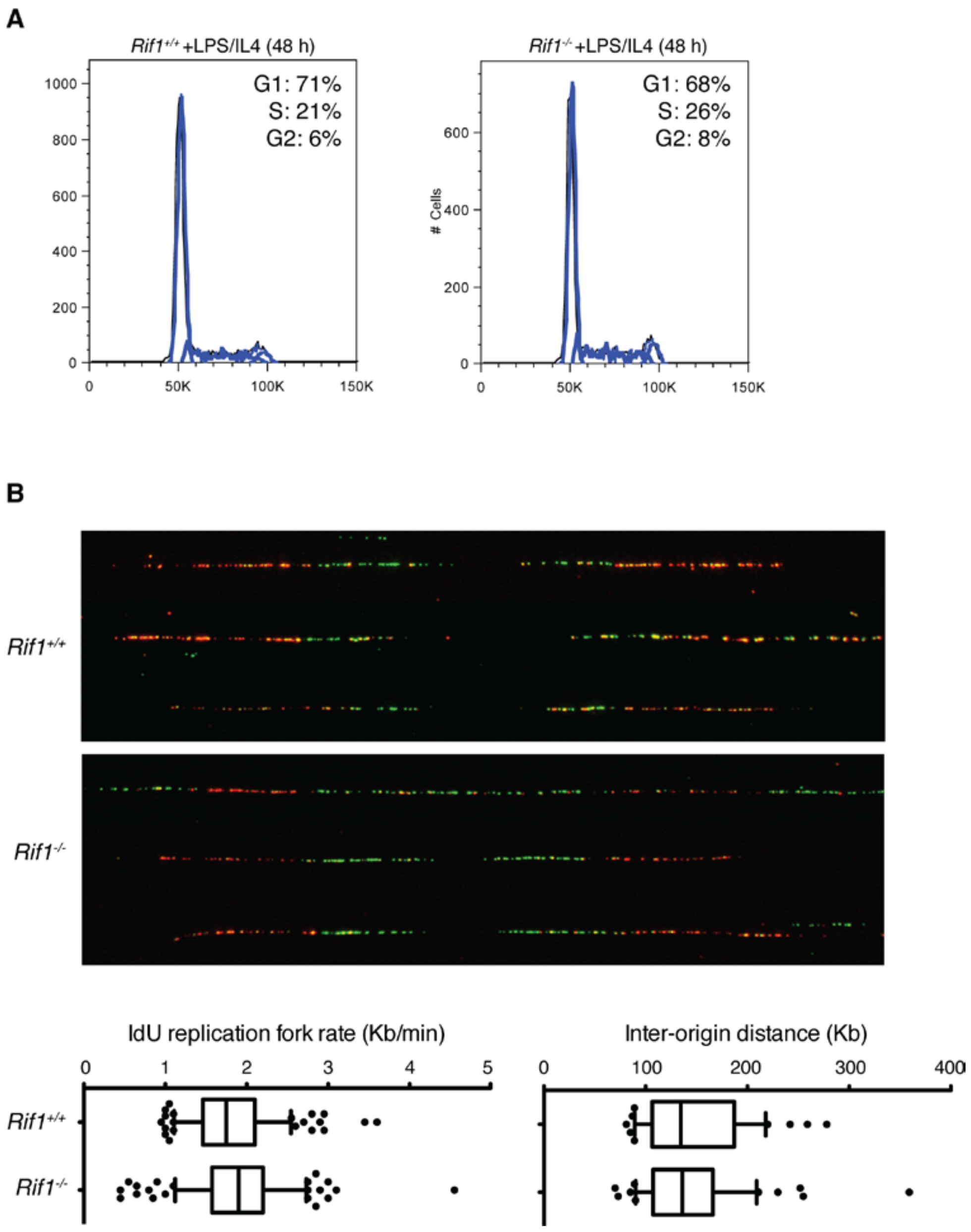

Figure S3 (related to Figure 1). Cell cycle profiles, replication fork rates and inter-origin distances in wild-type and RIF1-deficient cells

(**A**) Cell cycle analysis of stimulated splenic B-cells from mice of indicated genotype. Relative cell-cycle positions were calculated by flow cytometric analysis of DNA content (propidium iodide; presented) and BrdU incorporation (**B**) Replication fork rates and inter-origin distances in wild-type and Rif1-deficient early-passage primary MEFs. Shown are representative images of DNA fibers from cells of the indicated genotypes. Tracts labeled with IdU (green) CdU (red). Box-and-whiskers graphs are shown for IdU replication fork rates and inter-origin distances. The line line within the middle of the box marks the median (50th percentile) and the whiskers correspond to the 10–90 percentiles. Statistical analysis was performed with Prism v5.0 using the non-parametric Mann-Whitney rank sum test. 100 fibres were analysed per genotype to assess replication fork rates. 50 fibres were analysed per genotype to assess inter-origin distances.

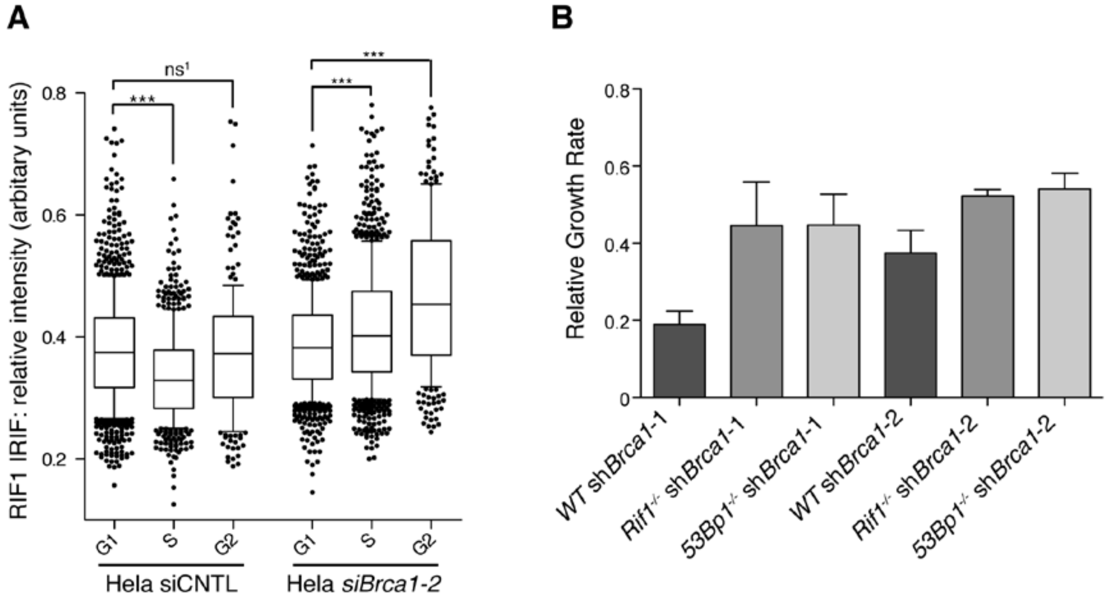

Figure S4 (related to Figure 3). Quantification of RIF1 IRIF intensity, Growth rates of *WT, Rif1*$^{-/-}$ and *53Bp1*$^{-/-}$ MEFs ± shBrca1

(**A**) RIF1 IRIF intensity measurements (as in Figure 3B and 3C) on cells subject to control or Brca1 depletion using a second transcript-targeting siRNA duplex. siBRCA1-2 cells analyzed, *n = 103*, *** *p < 0.0001*, ns[1] *p = 0.5511*, Mann–Whitney test. (**B**) Mean growth rates of indicated MEF lines were calculated from 4 day growth curves. Presented growth rates for each *Brca1*-targeting shRNA construct were normalized against growth rates of control shRNA-treated MEFs (control shRNA growth rate = 1) *n = 3* ± *SEM.*

A

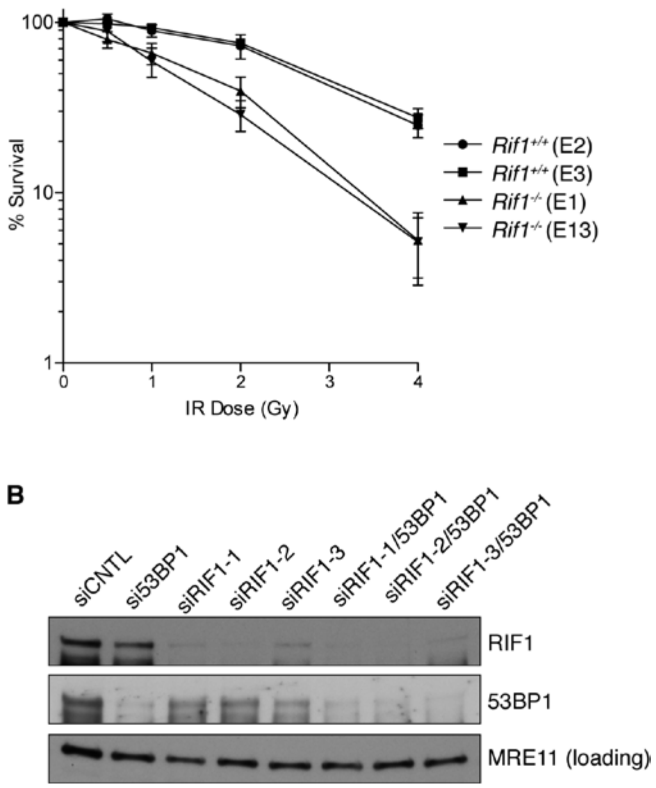

Figure S5 (related to Figure 4). IR sensitivity of *Rif1*$^{-/-}$ MEFs and western blots of siRNA knockdown in HEK293 EJ5 cells

(**A**) Multiple independently derived wild type and *Rif1*$^{-/-}$ MEF lines (outbred CD-1 derived) show similar sensitivities to ionizing radiation. Indicated MEF lines were subjected to colony survival assay as in Figure 4C. Error bars are ±SEM. (**B**) Western blotting shows typical RIF1 and 53BP1 protein-depletions achieved in experiments described in Figure 4D, using the indicated siRNA duplexes in HEK293 EJ5 cells.

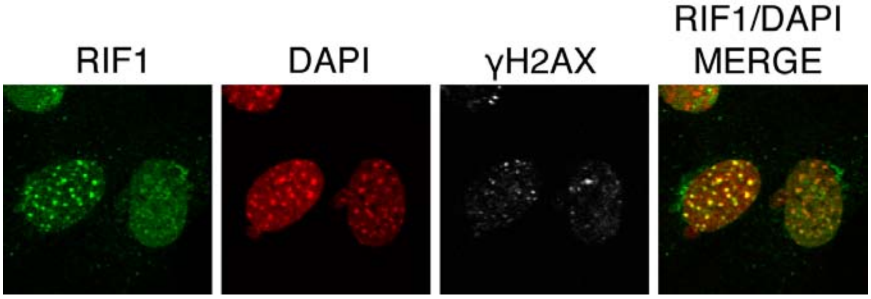

Figure S6 (related to Figure 6). DNA-damage independent enrichment of RIF1 in mouse heterochromatin domains
Fixed wild type MEFs were immunostained with RIF1 and γH2AX antisera and DNA counterstained with DAPI. DAPI is pseudo-colored in red to enable visualization of colocalisation in merge panel. Such RIF1 staining patterns account for the background RIF1 foci quantified in undamaged cells (Figure 6A).

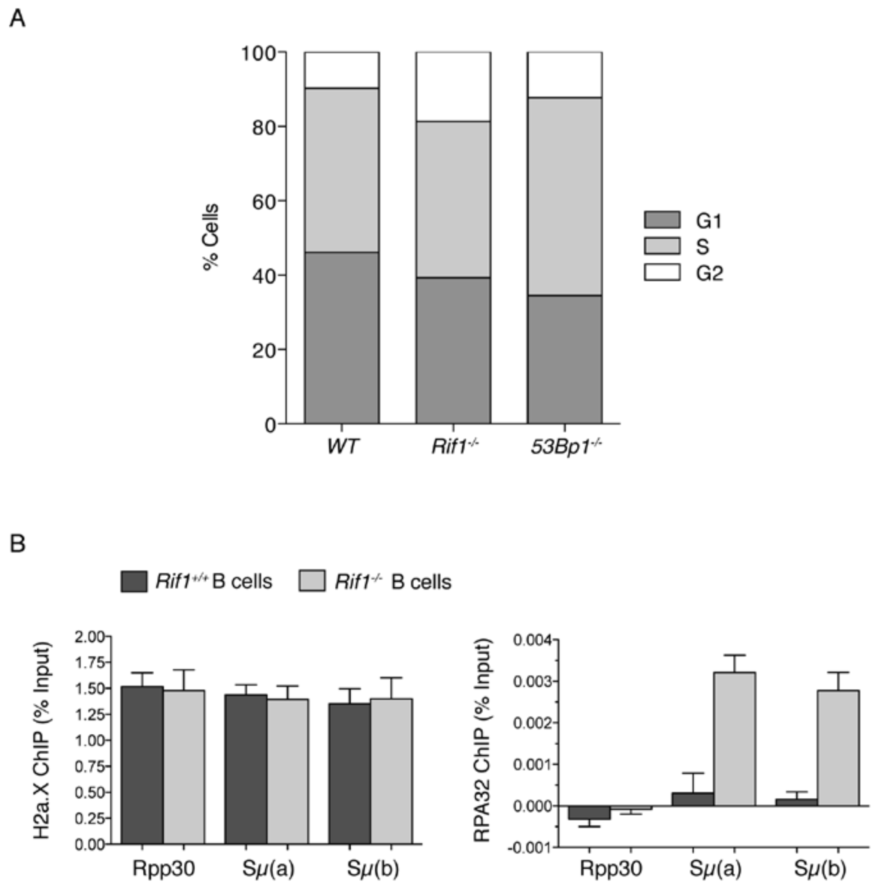

Figure S7 (related to Figure 7). Cell cycle profiles of WT *Rif1*$^{-/-}$ and *53Bp*$^{-/-}$ MEFs and ChIP lymphocyte data normalized against input chromatin

(**A**) MEF lines were pulse-labeled with BrdU (30 min, 10 µM) before fixation and DNA staining with propidium iodide. % cell-cycle phase distributions for each MEF line were calculated by flow cytometric analysis of DNA contenwts vs. BRdU fluorescence. (**B**) Background subtracted ChIP data presented in Figure 7E normalized against input chromatin. Mean ± SEM, n = 2 biological replicates.

A

Strain	Stage	*Rif1*$^{+/+}$	*Rif1*$^{+/-}$	*Rif1*$^{-/-}$
129/Ola	Blastocysts	7	13	3
	E7.5 embyros	4	12	5
	Live pups	6	16	0*
MF1	Live pups	31	73	0**

B

Strain	Stage		*Rif1*$^{+/+}$ ♂	*Rif1*$^{+/+}$ ♀	*Rif1*$^{+/-}$ ♂	*Rif1*$^{+/-}$ ♀	*Rif1*$^{-/-}$ ♂	*Rif1*$^{-/-}$ ♀
CD-1	Live pups	Obs:	35	38	71	59	15	0
	(218 screened)	Exp:	27	27	55	55	27	27
	Live pups (218),	Obs (%):	16	17	33	27	7	-
	♀ lethal corrected	Exp (%):	14	14	29	29	14	-

Table S1 (related to Figure 1). Frequency of viable *Rif1*$^{-/-}$ offspring in different genetic backgrounds
(A) Table of the genotypes found in the blastocysts, embryos, and live offspring of heterozygous intercrosses of *Rif1*$^{-}$ (XT278 genetrap) mice, in 129/Ola and outbred MF1 genetic backgrounds. * indicates significant deviation from Mendelian 1:2:1 ratio, $p<0.05$. ** indicates c^2-test $p<0.01$. (**B**) Table of the genotypes found in live pups from *Rif1*$^{+/-}$ intercrosses in outbred CD-1 genetic background. As RIF1-deficiency yielded female-specific lethality in CD-1 mice, sex of offspring is indicated. Obs, observed number of pups; and Exp, expected. (%) indicates corrected numbers accounting for sex-specific lethality. Genotypes were determined from embryo or blastocyst tissues, or from offspring at time of weaning. No evidence for postnatal lethality of *Rif1*-mutant pups in any background was observed.

Name	Sequences	Chromosome N^{o}. & sequence coordinates (NCBI37/mm9)
Sμ(a)	Fwd; CAATGTGGTTTAATGAATTTGAAGTTGCCA Rev; TCTCACACTCACCTTGGATCTAAGCACTGT	C12; 114664216-114664315
Sμ(b)	Fwd; GCTAAACTGAGGTGATTACTCTGAGGTAAG; Rev; GTTTAGCTTAGCGGCCCAGCTCATTCCAGT;	C12; 114663856-114663955
Rpp30	Fwd; TCCAGTGTGCAAGAAAGCTAAATG Rev; GGCAGTGCGTGGAGACTCA	C19; 36178902-36178963

Table S2. qPCR oligos used in IgH ChIP experiments

SUPPLEMENTAL EXPERIMENTAL PROCEDURES

Mice

Mice carrying the *Rif1*$^{-}$ allele were generated from an ES cell clone that contains a trapped *Rif1* allele (XT278, Sanger Institute Gene Trap Resource, Cambridge, UK). Chimeric mice were backcrossed 3 times into an outbred MF1 background, and twice into the 129/Ola strain. MF1 *Rif1*^{XT278} mice were later backcrossed three times into an outbred CD-1 background. The primers used for genotyping were XTf, 5'-GATCTCACTGTGCACTCCTCCATGG -3'; XTr, 5'-ACATAGTTGGCAGTGTTTGGGGCAAGTG-3'; and Wr, 5'-CTGGAAAGGTGGAGAAAGCAAGAACC-3'. A probe for non-radioactive Southern blotting was amplified by PCR from mouse genomic DNA using primers in exon 5 (5'-CAGACGTTTCCTGCTGAACTTGTTAGC-3') and intron 6 (5'-ATGCTTCAAAATCAACAACAGCAGAATG-3'), cloned into pBluescript II (Stratagene), labelled with digoxigenin by random priming, according to the supplier's instructions (Roche). *53Bp1* mice (Ward et al., 2003) were obtained from JAX® mice (Bar Harbor, ME, USA). Due to the background-specific viability of Rif1-deficient cells/mice, we were extremely careful to control for such differences in genetic backgrounds. In this regard, the WT, *Rif1*$^{-/-}$, *53Bp1*$^{-/-}$, and *Rif1*$^{-/-}$*53Bp1*$^{-/-}$ MEFs were all created from the embryos of double het matings, with the double knockout MEF line originating from the same female as the *53BP1*$^{-/-}$ line (E13.5), and WT and *Rif1*$^{-/-}$ lines obtained by a second timed mating of the same cohort and prepared the next day (E13.5). SV40 immortalization was then performed in parallel and experiments prepared in identical cell passages. Moreover, because Rif1-loss is female lethal, all MEF lines used in this study were derived from male embryos to ensure against sex-specific differences.

Antibodies, EdU labelling and Immunofluorescence

The following antibodies were used against mouse proteins in immunofluorescence (IF), western blot (WB), and chromatin-IP (ChIP) experiments: 53BP1 (NB-100-304, Novus; IF/WB), BRCA1 C17 (a gift from A. Nussenzweig (NCI, Bethesda, MD, USA; WB)), CHK1 (C9358, Sigma; WB), CHK1 pSer345 (133D3, Cell Signaling Technology; WB), CHK2 (05-649, Millipore; WB), H2a.X (3522-1, Epitomics; WB/ChIP), HA.11 (16B12, Covance; IF/WB), RPA32 (12F3.3, Abcam; WB), RPA34-20 (Ab-3, Calbiochem; ChIP), γTubulin (GTU-88, Abcam; WB), SMC1 (AB21583, Abcam; WB). Affinity-purified rabbit polyclonal antibodies SK1315 (IF/WB) and SK1316 (IF/WB) raised against mouse Rif1 were previously described (Adams and McLaren, 2004). In addition, Cyclin A (clone AT12, a gift from Julian Gannon, CRUK, UK), Rif1 (A300-569A, Bethyl; IF) and BRCA1 (D9, Santa Cruz; IF/WB) were used to stain human proteins. Nucleotide incorporation was measured by EdU pulse-labelling (5 min at 40 μM), and was labelled in fixed cells under native conditions using the Click-IT EdU 594-Alexa Fluo Labeling Kit (Molecular Probes) according to manufacturers instructions. Indirect immunofluorescence was performed on an Olympus FLV1000 inverted microscope essentially as in (Chapman et al., 2012), yet for visualization of RIF1, paraformaldehyde (2%) fixed cells were permeabilized in 0.2% Triton X-100 before blocking and subsequent immunostaining. Automated IRIF counting and IRIF intensity measurements was performed using Cell Profiler software (Carpenter et al., 2006). Briefly, focal volumes were identified, counted, and assigned to parent nuclei. Mean focal intensities were calculated for individual foci, and parent nuclus cell cycle phases assigned through intensity measurements of EdU and Cyclin A signals.

Cell culture and transfection

SV40 transformed MEFs prepared from E13.5 embyos and cultured in DMEM + 10% FCS + Pen/Strep were employed in experiments unless otherwise stated. Cells were incubated with ATM inhibitors (10 µM) at least 30 min before irradiation. Olaparib (Axon Biochemicals BV) was used as stated. Metaphase spreads were prepared as previously described (Wechsler et al., 2011). MEFs were incubated for 90 min with colcemid prior to harvesting metaphase cells. MEFs were swollen in 0.075 M KCl for 20 min then fixed in methanol/acetic acid (3:1) prior to spreading. For radial chromosomes counting, metaphase spreads were stained in 7% Giemsa solution for 6min prior to extensive washes with bidest water. FISH was performed using a FITC-(CCCTAA)3 PNA probe (Bio Synthesis). DNA was counterstained with 4,6-diamidino-2-phenylindole (DAPI) before the last PBS wash and addition of Vectashield mounting media (Vector). Digital images were captured with Zeiss Axio Imager M1 microscope. 50 independent metaphase spreads were taken randomly from each condition in order to quantify the respective phenotypes. Olaparib was purchased from Axon Medchem. Vector and siRNA transfections were performed using XtremeGene-HP (Roche) and siRNA-MAX (Invitrogen) reagents, respectively, according to manufacturers instructions. siRNA oligos were as follows:

- RIF1-1: 5'- GCA UUG ACU UCU CAC CAU A-3'
- RIF1-3: 5'-CGU AGA GAU UAG UGA AAC A-3'
- RIF1-6: 5'-AAG AGC AUC UCA GGG UUU GCU-3'
- 53BP1: 5'-GAA GGA CGG AGU ACU AAU A-3'
- BRCA1-1: Dharmacon Smartpool MU-003461-01; 5'-CAG CUA CCC UUC CAU CAU AUU-3'; 5'-GGG AUA CCA U GC AAC AUA AUU-3'; 5'-GAA GGA GCU UUC AUC AUU CUU-3'; 5'-CUA GAA AUC UGU UGC UAU GUU-3'
- BRCA1-2: 5'-GCA GUG AAG AGA UAA AGA A-3'
- CONTROL (LUCIFERASE): 5'-CGU ACG CGG AAU ACU UCG ATT-3'

Retrovirus/Lentivirus-mediated delivery of expression and shRNA constructs

pLPC-Myc-TRF2 and pLPC-Myc-TRF2$^{\Delta B\Delta M}$ retroviral constructs were used as previously described by (van Steensel et al., 1998). Briefly, retroviral constructs were transfected in AmphoPack 293 cells. After 2 days, supernatant (SN) was collected successively for 3 days. Selected genotyped MEFs were infected with 4 μg of polybrene/ml for 24h. After 3 days of infection, the cells were selected with 3μg of puromycin/ml. *Brca1*-targeting shRNA oligos 1 (target seq: CCAAGAAGAGGATAGTATAAT) and 2 (target seq: GTGCTTCCACACCCTACTTAC) were cloned into pLKO.1-Puro plasmid (Addgene plasmid 10878), and these or a control (empty vector) were used to generate lentivirus to deliver shRNA. Viral SNs were prepared as described on the Addgene website, and frozen SNs used to transduce MEFs in two sequential infections 12 h apart. MEFs were then selected in 3μg/ml puromycin 24 h following first infection and experiments were typically performed from 72 h onwards. 53BP1 constructs cloned into the pHAGE N-Flag-HA vector were co-transfected into HEK293T cells with the HDM-Tat1b, HDM-VSV-G, HDM-Hgpm2 and pRC-cmvRev1b viral packaging vectors. 48 and 60 h viral supernatants were collected and used to transduce MEF lines. MEFs were subsequently selected and maintained in 3 and 1μg/ml concentrations of puromycin, respectively.

Chromatin Immunoprecipitation

B cell suspensions were fixed with 0.75% (v/v) formaldehyde (28908,Thermo) at room temperature for 10 min and quenched with 20 mM glycine for 5 min. Cells were rinsed two times with 10 ml of cold PBS. Cell lysed in ChIP lysis buffer (1% SDS, 10 mM EDTA, 50 mM Tris pH8, +protease inhibitors (PI; complete, Roche)) at 4 x 10^7 per ml concentration before chromatin shearing in a sonicating waterbath (Bioruptor, Diagenode) to obtain mean fragment size of between 200-600 bp. Subsequently, sonicates were clarified by centrifugation, diluted 10-fold in ChIP dilution buffer (1.1%

Triton X-100, 1.2 mM EDTA, 16.7 mM Tris pH 8, 167 mM NaCl). 5% input samples were taken at this stage and ChIPs perform in lysates derived from 10^7 cells for 4 h using 2-5 µg antibody pre-conjugated to 40 µl Protein G Dynabeads (Dynal, Invitrogen) for 4 h. ChIPs were subsequent washed 2 x in TSE-150 (1% Triton X-100, 0.1% SDS, 2mM EDTA, 20 mM Tris pH 8, 150 mM NaCl), 1 x in TSE-500 (0.1% SDS, 1%, Triton X-100, 500mM NaCl, 2mM EDTA, 20mM Tris-HCl, pH 8), 1 x in LiCl buffer (0.25M LiCl, 1% (v/v) NP-40, 1% (v/v) DOC, 1mM EDTA, 10mM Tris, pH 8) and 1 x in TE (10 mM Tris-HCl, pH8, 0.1 mM EDTA). Immuno-complexes were eluted from the beads with a total of 125 µl of elution buffer (100 mM sodium bicarbonate, 1% (v/v) SDS) for 30 min at 30°C and de-crosslinked overnight at 65°C. De-crosslinked ChIP and Input samples were purified with the Qiaquick® PCR purification kit (Qiagen) and DNA was eluted from the columns with 50 µl of water. From each immunoprecipitation, 1-5 µl of eluted sample was used in Real-time PCR analysis in triplicate on an CFX96 Real-Time Analyzer (Biorad) with the use of iQ SYBR® Green (Biorad) for each primer pair (see Table S2).

Lymphocyte analyses and flow cytometry

Lymphocyte populations were analysed by flow cytometry in single cell suspensions from spleen, thymus and bone marrow. For staining, anti-mouse CD16/32 (2.4G2, BD Biosciences) was used to block non-specific antibody binding. Afterwards the following directly labelled anti-mouse antibodies were used: anti-mouse B220-APC-Cy7 (RA3-6B2), CD43-FITC (S7), CD24-PE (M1/69), BP-1-biotin (6C3), IgM-PECy7 (R6-60.2), CD21-FITC (7G6), CD4-APC (RM4-5; all from BD Biosciences), IgD-APC (11-26), TCR-b-PE (H57-597; eBiosciences), CD8-PE (53-6.7; Biolegend) and CD23-APC (2G8; SouthernBiotech); followed by streptavidin eFluor 450 (eBiosciences) when biotinylated antibodies were used. Data were collected on an LSRFortessa flow cytometer (Becton Dickinson) and were analysed with FlowJo software (TreeStar).

Purification of splenic B cells and in vitro CSR

B cells were purified by negative selection from single cell suspensions from spleen using magnetic separation B cell isolation kit (Miltenyi Biotec) according to manufacture's instructions. Purified cells (purity>95%) were cultured for 3 to 6 days at 10^6 cells/ml in RPMI supplemented with 10%FCS and LPS (10 µg/ml, Sigma), IL-4 (10 ng/ml, R&D) and/or anti-mouse CD40 antibody (5 µg/ml; FGK45, Enzo Life Sciences). For proliferation analyses B cells were labelled with 5 mM Cell Trace Violet (Molecular Probes) in PBS for 15 min at 37°C before culture. For flow cytometry analyses of CSR cells were resuspended in PBS with 2% BSA and were stained with anti-mouse CD19-APC (1D3, BD Biosciences), goat anti-mouse IgG1-FITC (SouthernBiotech) and goat anti-mouse IgG2b-Alexa Fluor 546 (Invitrogen). For viability analyses B cells were stained with Annexin V-FITC (BD Biosciences) and propidium iodide (PI) for 15 min at room temperature before flow cytometry analyses.

Immunizations

Mice were immunized intraperitoneally with 50 mg of NP-KLH (Biosearch Technologies) emulsioned in Imject Alum adjuvant (Pierce). Blood samples were collected from the tail-vein at 0, 7, 14, 21 and 28 days after immunization.

ELISA

Enzyme-linked immunosorbant assays (ELISAs) were used to quantify the production of antigen-specific antibodies in mice serum. Briefly, 96 well plates were coated with 1 µg/ml NP-BSA (Biosearch Technologies) in PBS, blocked with PBS 2.5% FCS and incubated with serial dilutions of serum collected at different time points from immunized mice. Plates were probed using biotinylated antibodies against mouse IgM, IgG, IgG_1, IgG_{2b} (Southern Biotech) and IgG3 (BD Biosciences) followed by streptavidine-alkaline phosphatase and p-Nitrophenyl phosphate as substrate for detection (Sigma). Optical density was measured at 405 nm. Total

antibody levels in mice sera or culture supernatants were determined by sandwich ELISA. Total IgG_1 and IgG_{2b} were measured with mouse IgG_1 and IgG_{2b} ELISA kit respectively (eBiosciences) according to the manufacturer's instructions. For analyses of total IgM, IgG_3 and IgA anti-mouse IgM (II/41), IgG_3 (R2-38) and IgA (C10-3, all from BD Biosciences) were used for capture and biotinylated anti-mouse IgM (R6-60.2), IgG_3 (R40-82, both BD Biosciences) and IgA (11-44-2, eBioscences) for detection. Purified mouse IgM (BD Biosciences), IgG_3 and IgA (Southern Biotech) were used as standard.

NHEJ reporter assays

DSB repair efficiency by NHEJ was measured in EJ5-GFP HEK293 cell lines as described previously (Bennardo et al., 2008). In brief, 48 h after siRNA transfection, cells either mock-transfected or transfected with 0.6 µg of an I-SceI expression plasmid (pCBASce) using 1.2 µl of JetPrime (Polyplus). 4 h after I-SceI transfection, the media was replaced, followed by a second transfection with siRNA aligos (15nM). 48 h after after I-SceI transfection, cells were analysed for GFP expression by flow cytometry on a Cyan ADP (Dako).

DNA combing

DNA combing was performed as described in (Michalet et al., 1997). Briefly, early passage of primary cells of the indicated genotype were pulse-labeled with IdU/CldU for 30min, each pulse. DNA fibres were extracted in agarose plugs and stretched on silanized coverslips with the molecular combing system (Genomic Vision). CldU was detected with Rat anti-BrdU antibody (BU1/75, AbCys; 1/20), followed by Goat anti-Rat coupled to Alexa 594 (A11007, Molecular Probes; 1/50) and finally by Chicken anti-Goat coupled to Alexa 594 (A21468, Molecular Probes; 1/50). IdU was detected with Mouse anti-BrdU coupled to FITC antibody (BD44, Becton Dickinson; 1/20), followed by Rabbit anti-Mouse coupled to Alexa 488 (A11059, Molecular Probes;

1/50) and finally by Donkey anti-Rabbit coupled to Alexa 488 (A21206, Molecular Probes; 1/50). DNA fibres were captured with Zeiss Axio Imager M1 microscope equipped with an ORCA-ER camera (Hamamatsu) controlled by Volocity 4.3.2 software (Improvision). Representative images of DNA fibres were assembled from different fields of view and were processed as described in (Michalet et al., 1997).

Microscopy

Indirect immunofluorescence and cell-cycle analysis was performed as described previously (Chapman et al., 2012). Automated IRIF counting and intensity measurements were performed using Cell Profiler software (Broad Institute), or scored manually (see also Supplementary Information).

SUPPLEMENTAL REFERENCES

Adams, I.R., and McLaren, A. (2004). Identification and characterisation of mRif1: a mouse telomere-associated protein highly expressed in germ cells and embryo-derived pluripotent stem cells. Dev Dyn *229*, 733-744.

Bennardo, N., Cheng, A., Huang, N., and Stark, J.M. (2008). Alternative-NHEJ is a mechanistically distinct pathway of mammalian chromosome break repair. PLoS Genet *4*, e1000110.

Carpenter, A.E., Jones, T.R., Lamprecht, M.R., Clarke, C., Kang, I.H., Friman, O., Guertin, D.A., Chang, J.H., Lindquist, R.A., Moffat, J.*, et al.* (2006). CellProfiler: image analysis software for identifying and quantifying cell phenotypes. Genome Biol *7*, R100.

Chapman, J.R., Sossick, A.J., Boulton, S.J., and Jackson, S.P. (2012). BRCA1-associated exclusion of 53BP1 from DNA damage sites underlies temporal control of DNA repair. J Cell Sci *125*, 3529-3534.

Michalet, X., Ekong, R., Fougerousse, F., Rousseaux, S., Schurra, C., Hornigold, N., van Slegtenhorst, M., Wolfe, J., Povey, S., Beckmann, J.S.*, et al.* (1997). Dynamic molecular combing: stretching the whole human genome for high-resolution studies. Science *277*, 1518-1523.

van Steensel, B., Smogorzewska, A., and de Lange, T. (1998). TRF2 protects human telomeres from end-to-end fusions. Cell *92*, 401-413.

Ward, I.M., Minn, K., van Deursen, J., and Chen, J. (2003). p53 Binding protein 53BP1 is required for DNA damage responses and tumor suppression in mice. Molecular and cellular biology *23*, 2556-2563.

Wechsler, T., Newman, S., and West, S.C. (2011). Aberrant chromosome morphology in human cells defective for Holliday junction resolution. Nature *471*, 642-646.
