# Supplementary material for: RIF1 Is Essential for 53BP1-Dependent Nonhomologous End Joining and Suppression of DNA Double-Strand Break Resection
Source: Mol Cell. 2021 Jul 1;81(13):2868. doi: 10.1016/j.molcel.2021.06.015 (PMC8260204; doi:10.1016/j.molcel.2021.06.015)
Supplement: Document S1. Details of correction to Figure 6E, including scans of original X-ray films [file mmc1.pdf]

Molecular Cell, Volume *81*

Supplemental information

RIF1 Is Essential for 53BP1-Dependent Nonhomologous End Joining and Suppression of DNA Double-Strand Break Resection

J. Ross Chapman, Patricia Barral, Jean-Baptiste Vannier, Valérie Borel, Martin Steger, Antonia Tomas-Loba, Alessandro A. Sartori, Ian R. Adams, Facundo D. Batista, and Simon J. Boulton

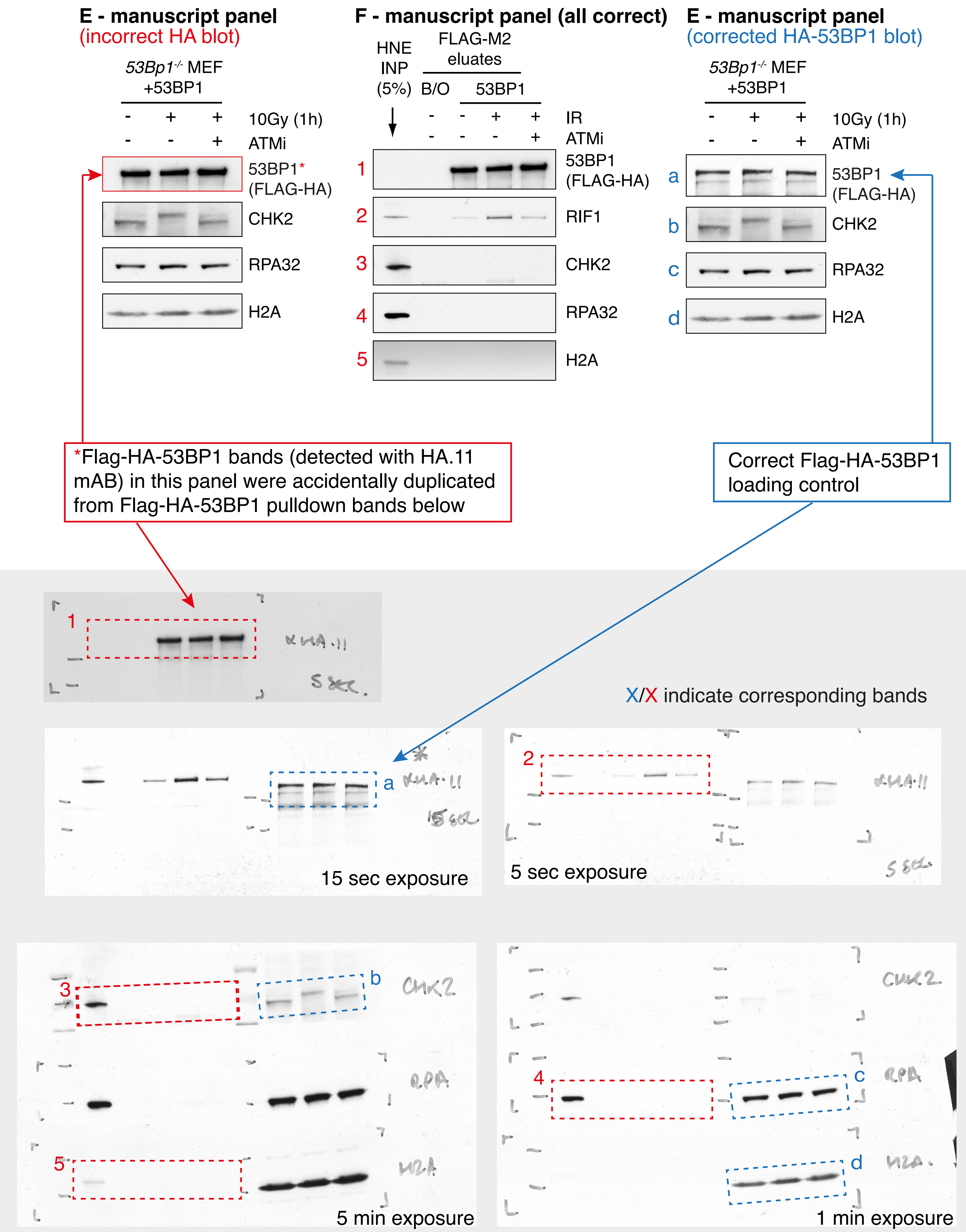

Scans of x-ray films archived from November 2012; new scans performed on April 9th 2021
