# Supplementary material for: RIF1 Is Essential for 53BP1-Dependent Nonhomologous End Joining and Suppression of DNA Double-Strand Break Resection
Source: Mol Cell. 2021 Jul 1;81(13):2868. doi: 10.1016/j.molcel.2021.06.015 (PMC8260204; doi:10.1016/j.molcel.2021.06.015)
Supplement: Document S2. Article plus supplemental information [file mmc2.pdf]

Molecular Cell

CellPress
OPEN ACCESS

Correction

RIF1 Is Essential for 53BP1-Dependent Nonhomologous End Joining and Suppression of DNA Double-Strand Break Resection

J. Ross Chapman, Patricia Barral, Jean-Baptiste Vannier, Valérie Borel, Martin Steger, Antonia Tomas-Loba, Alessandro A. Sartori, Ian R. Adams, Facundo D. Batista, and Simon J. Boulton*
*Correspondence: simon.boulton@cancer.org.uk
https://doi.org/10.1016/j.molcel.2021.06.015

(Molecular Cell *49*, 858–871; March 7, 2013)

In the original published version of this article, the *53BP1 (Flag-HA)* loading control HA immunoblot panel in Figure 6E was an accidental duplication of the *53BP1 (Flag-HA)* pulldown HA immunoblot bands in Figure 6F. This mistake arose during figure preparation and was possible since both blots originated from a single experiment (resolved on common X-ray films), consistent with what is stated in the figure legend. The correct *53BP1 (Flag-HA)* loading control HA immunoblot from Figure 6E is displayed here. The error does not alter the results or conclusions that can be drawn from this experiment or any other in this manuscript. The authors regret this error.

SUPPLEMENTAL INFORMATION

Supplemental information can be found online at https://doi.org/10.1016/j.molcel.2021.06.015.

E

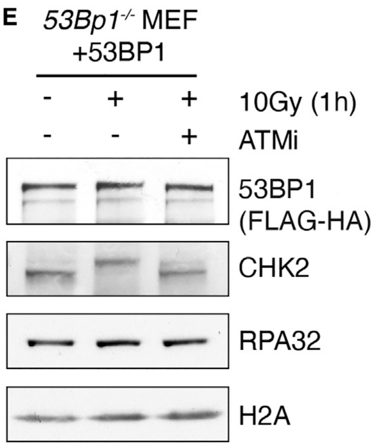

Figure 6E. ATM-Dependent Phosphorylation of 53BP1 Promotes RIF1 Interaction

 © 2021 Elsevier Inc.
This is an open access article under the CC BY IGO license (http://creativecommons.org/licenses/by/3.0/igo/).

Molecular Cell, Volume *81*

Supplemental information

RIF1 Is Essential for 53BP1-Dependent Nonhomologous End Joining and Suppression of DNA Double-Strand Break Resection

J. Ross Chapman, Patricia Barral, Jean-Baptiste Vannier, Valérie Borel, Martin Steger, Antonia Tomas-Loba, Alessandro A. Sartori, Ian R. Adams, Facundo D. Batista, and Simon J. Boulton

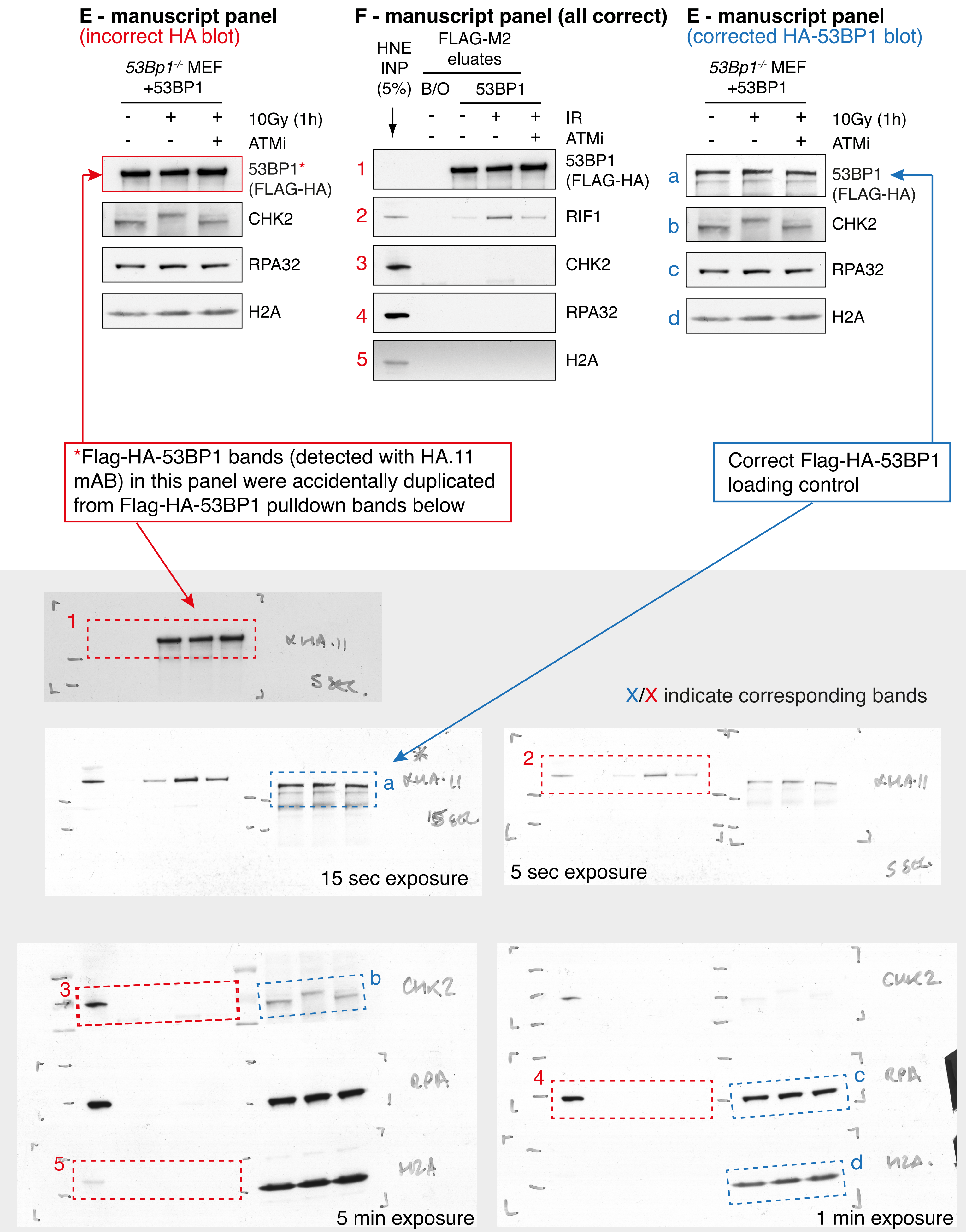

Scans of x-ray films archived from November 2012; new scans performed on April 9th 2021
